# Self-organized optofluidic locking enables stable single-frequency lasing in dynamic liquid media

**DOI:** 10.1126/sciadv.aei3045

**Published:** 2026-09-23

**Authors:** Rui Duan, Yuan Wang, Bojian Shi, Yaqi Tang, Lin Wang, Yan Wang, Bei Xu, Junzi Li, Yichen He, Kailei Lu, Yanli Shi, Xiaoyan Zhou, Lin Zhang, Hongchang Deng, Tingchao He, Xuyong Yang, Rui Chen, Jianqi Qi, Huiyun Liu, Handong Sun

**Affiliations:** ^1^ College of Physics, Sichuan University, Chengdu 610064, China.; ^2^ Macao Centre for Research and Development in Advanced Materials, Institute of Applied Physics and Materials Engineering, University of Macau, Taipa, Macao SAR 999078, China.; ^3^ Institute of Advanced Photonics, School of Physics, Harbin Institute of Technology, Harbin 150001, China.; ^4^ Guangxi Key Laboratory of Optoelectronic Information Processing, Guilin University of Electronic Technology, Guilin 541004, China.; ^5^ Key Laboratory of Advanced Display and System Applications of Ministry of Education, Shanghai University, Shanghai 200072, China.; ^6^ School of Electronics and Information, Academy for Quantum Science and Technology, Zhengzhou University of Light Industry, Zhengzhou 450001, China.; ^7^ State Key Laboratory of Radio Frequency Heterogeneous Integration, Key Laboratory of Optoelectronic Devices and Systems of Ministry of Education and Guangdong Province, College of Physics and Optoelectronic Engineering, Shenzhen University, Shenzhen 518060, China.; ^8^ School of Precision Instruments and Optoelectronics Engineering, Tianjin University, Tianjin 300072, China.; ^9^ Department of Physics, Faculty of Science, University of Macau, Macao SAR 999078, China.; ^10^ Department of Electronic and Electrical Engineering, University College London, Torrington Place, London WC1E 7JE, UK.

## Abstract

Liquid-state lasers are promising wavelength-agile, reconfigurable coherent light sources, but fluid gain media introduce Brownian motion, thermal transport, and refractive-index fluctuations that destabilize single-frequency emission. Here, we demonstrate that nonequilibrium fluid fluctuations can instead be harnessed to stabilize lasing through optofluidic locking. In anisotropic colloidal nanoplatelets (CNPs), a weak auxiliary continuous-wave optical field induces collective particle migration, reorientation, and accumulation via coupled optical forces and convective flow. The resulting self-organized gain landscape suppresses cavity fluctuations, improves output stability by nearly three orders of magnitude, and reduces the lasing threshold by over 75%. Integrated with high-gain CdSe/CdSeS/CdZnS core/graded-crown/graded-shell nanoplatelets and a Littman-like external cavity, this mechanism enables stable single-frequency lasing with a side-mode suppression ratio above 31 dB, a 0.051 nm linewidth, and 170-nm single-mode tunability across engineered CNP gain media. We further demonstrate applications in WS_2_ photoluminescence pumping and wavelength-tunable topological vertical-cavity lasing.

## INTRODUCTION

High-spectral-purity lasers are indispensable for precision metrology, quantum control, and ultrasensitive detection, where frequency noise and linewidth fundamentally limit measurement accuracy (*1*–*3*). In this context, liquid lasers are particularly attractive because liquid gain media can provide broad gain bandwidth, wide wavelength tunability, and efficient heat dissipation through convection or flow under high-power pumping (*4*). These unique features make them promising candidates for tunable, high-power, and reconfigurable coherent light sources. Nevertheless, the spectral performance of liquid lasers is strongly affected by the intrinsic dynamic nature of liquid media. Unlike dopant ions in solid-state crystals, active molecules in liquids undergo continuous Brownian motion, giving rise to microscopic density fluctuations and consequently local refractive-index variations. These refractive-index fluctuations perturb the intracavity optical path length and induce shifts of the cavity-mode frequencies. As a result, the longitudinal modes exhibit enhanced frequency jitter and mode wandering, leading to linewidth broadening and degraded frequency stability (*5*). More broadly, these coupled stochastic and thermally driven fluctuations make it fundamentally difficult to sustain a spectrally pure and stable single-frequency lasing state in liquid gain media. These effects severely compromise the spectral purity and coherence of liquid lasers, thereby limiting their practical use in applications requiring highly stable and narrow-linewidth laser emission.

To overcome this barrier, researchers have continuously explored novel gain media and resonator control schemes. In recent years, colloidal nanoplatelets (CNPs) have shown tremendous potential in the laser field due to their unique structure and physical properties. Compared to traditional colloidal quantum dots (CQDs), CNPs exhibit strong quantum confinement in the vertical direction, leading to ultranarrow emission linewidths (*6*–*8*), giant oscillator strength (*9*, *10*), larger optical gain coefficients (*11*, *12*), and suppressed nonradiative Auger recombination (*13*, *14*), making them ideal candidates for next-generation laser gain media. While considerable progress has been made in optically pumped CNP lasers (*6*, *7*, *15*–*26*), high-coherence operation in liquid-phase colloidal lasers remains elusive because improvements in gain media alone do not eliminate the dynamic instability of the liquid cavity itself (*27*–*28*). Compared to mature technologies such as dye-based liquid lasers and semiconductor laser diodes, colloidal nanocrystal liquid lasers remain largely at the proof-of-concept stage, with several key issues that must be addressed before practical applications can be realized: (i) Low side-mode suppression ratio (SMSR). Commercial lasers typically achieve an SMSR >30 dB (with a signal-to-side-mode ratio of at least 1000:1), while current liquid nanocrystal lasers show only 10 to 15 dB, far below the practical threshold. (ii) Limited precise wavelength tuning. Conventional Littrow-type external cavity configurations offer narrow tuning ranges and suffer from mode hopping, an issue that is largely suppressed in commercial tunable lasers. (iii) Pronounced output power fluctuations. Radiation pressure from high-peak-power pulsed pumping dynamically disturbs the spatial distribution of the nanocrystal gain medium, making it difficult to maintain stable output. Together, these limitations point to a central unresolved challenge: how to convert the intrinsically dynamic nature of liquid gain media from a source of decoherence into a mechanism for stabilizing coherent emission.

This work proposes an optofluidic self-stabilization mechanism that leverages the structural anisotropy of CNPs to address these challenges. Rather than suppressing fluidic dynamics externally, we show that they can be converted through a self-organized optofluidic locking process into a stabilizing mechanism for coherent emission. Under the combined action of optical gradient and scattering forces, CNPs in the liquid medium exhibit collective migration, reorientation, and accumulation within the optically favored region, thereby suppressing output fluctuations and markedly improving spectral purity. In addition, core/graded-crown/graded-shell CNPs are used as the gain medium. Their substantially extended gain lifetime arises from efficient hot-carrier cooling, multiexciton processes, and effective mitigation of the hot-phonon bottleneck, which together facilitate rapid transfer from high-energy multiexciton states and favor the establishment of a stable population inversion. By integrating this optofluidically locked gain medium with a tunable external cavity, we access a stable single-frequency lasing regime with improved spectral purity, suppressed intensity noise, and expanded wavelength agility. These results establish optofluidic locking as a general strategy for stabilizing liquid-state lasers and suggest a broader route toward high-coherence photonic functionality in dynamically reconfigurable soft-matter systems.

## RESULTS

### Overview of optofluidic locking enables CNP liquid laser

**Fig. 1.**
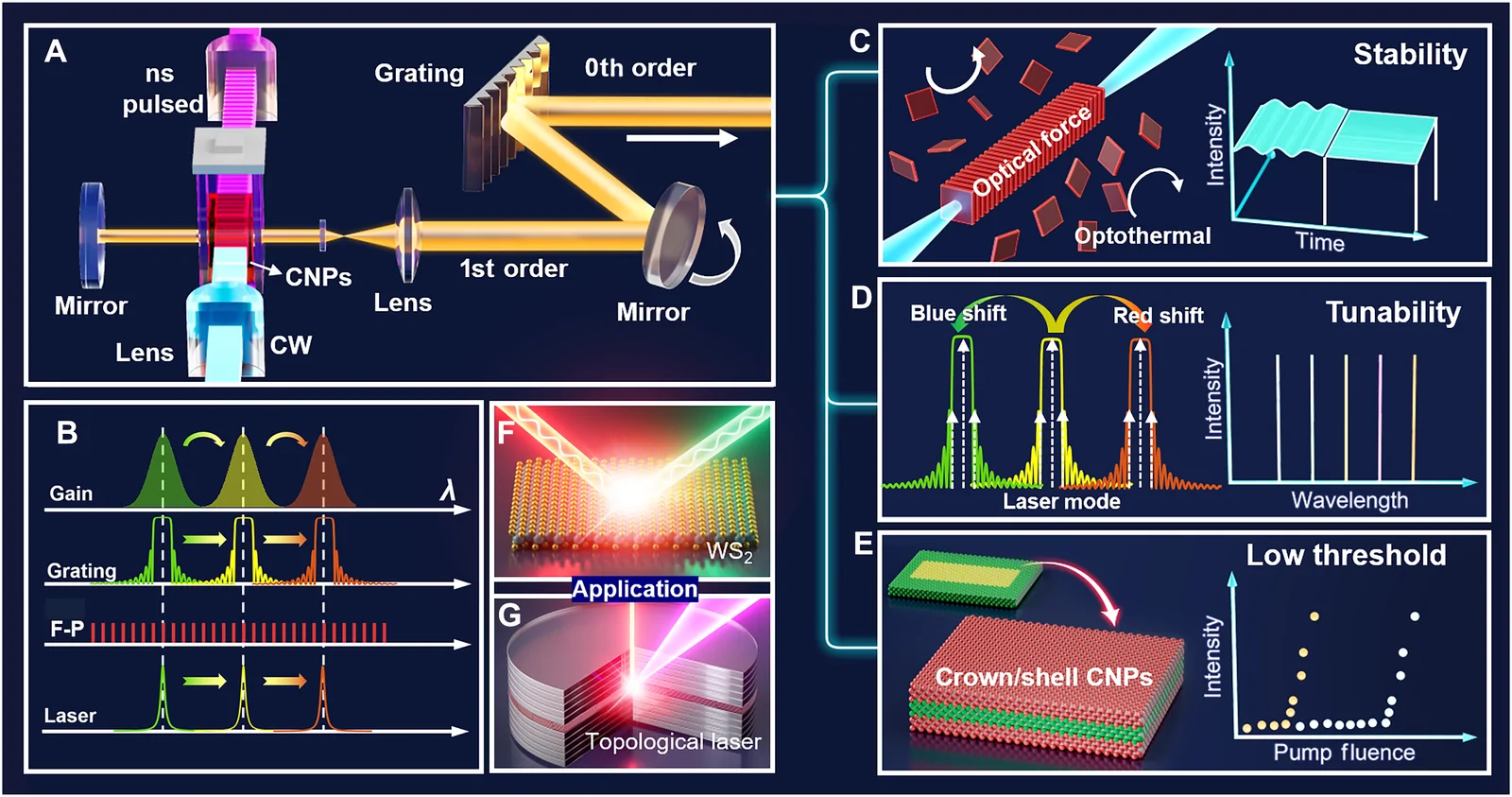
Design concept and representative applications of the optofluidically stabilized CNP liquid laser. (**A**) Schematic of the modified Littman-like external-cavity laser incorporating optofluidic locking. (**B**) Illustration of the Vernier-effect-enabled single-mode lasing mechanism arising from the interplay among the gain spectrum, grating response, and cavity resonances. (**C**) Schematic of the optofluidic locking mechanism, in which optical gradient and scattering forces, together with thermally induced convective flow, redistribute and stabilize the CNP gain medium. (**D**) Principle of wavelength tuning: Synchronous variation of the cavity length and incidence angle enables controlled blue and red shifts of the single-mode emission over a broad spectral range. (**E**) Structure of the CdSe/CdSeS/CdZnS core/graded-crown/graded-shell CNPs, designed to enhance optical gain and support low-threshold lasing. (**F** and **G**) Representative applications of the liquid laser in exciting the PL of monolayer WS_2_ and pumping a topological vertical-cavity laser.

This study implements a modified Littman-like external cavity in which a rotating mirror replaces the conventional rotating grating, as illustrated in Fig. 1A. Compared with linear displacement actuators (e.g., piezoelectric transducers), the rotating mirror offers a large tuning range with minimal angular excursion and high repeatability. Single-mode operation is achieved via a Vernier effect arising from the interplay among the gain spectrum, the grating response, and the Fabry-Pérot resonances formed between the grating and the front mirror (Fig. 1B). To enhance output stability, a continuous-wave light field is used to optically “lock” the CNPs (Fig. 1C). Rather than acting merely as an auxiliary trapping field, this optical input triggers a self-organized optofluidic locking process that dynamically redistributes the anisotropic CNP gain medium and suppresses cavity fluctuations. This design therefore couples cavity selection with gain-medium self-stabilization, providing a physical route toward stable single-frequency lasing in a dynamic liquid environment. Moreover, by synchronously varying the cavity length and the incidence angle through the rotating mirror, the system enables single-mode wavelength tuning across a broad spectral range (Fig. 1D).

### Optical properties of CdSe/CdSeS/CdZnS core/crown/shell CNPs

**Fig. 2.**
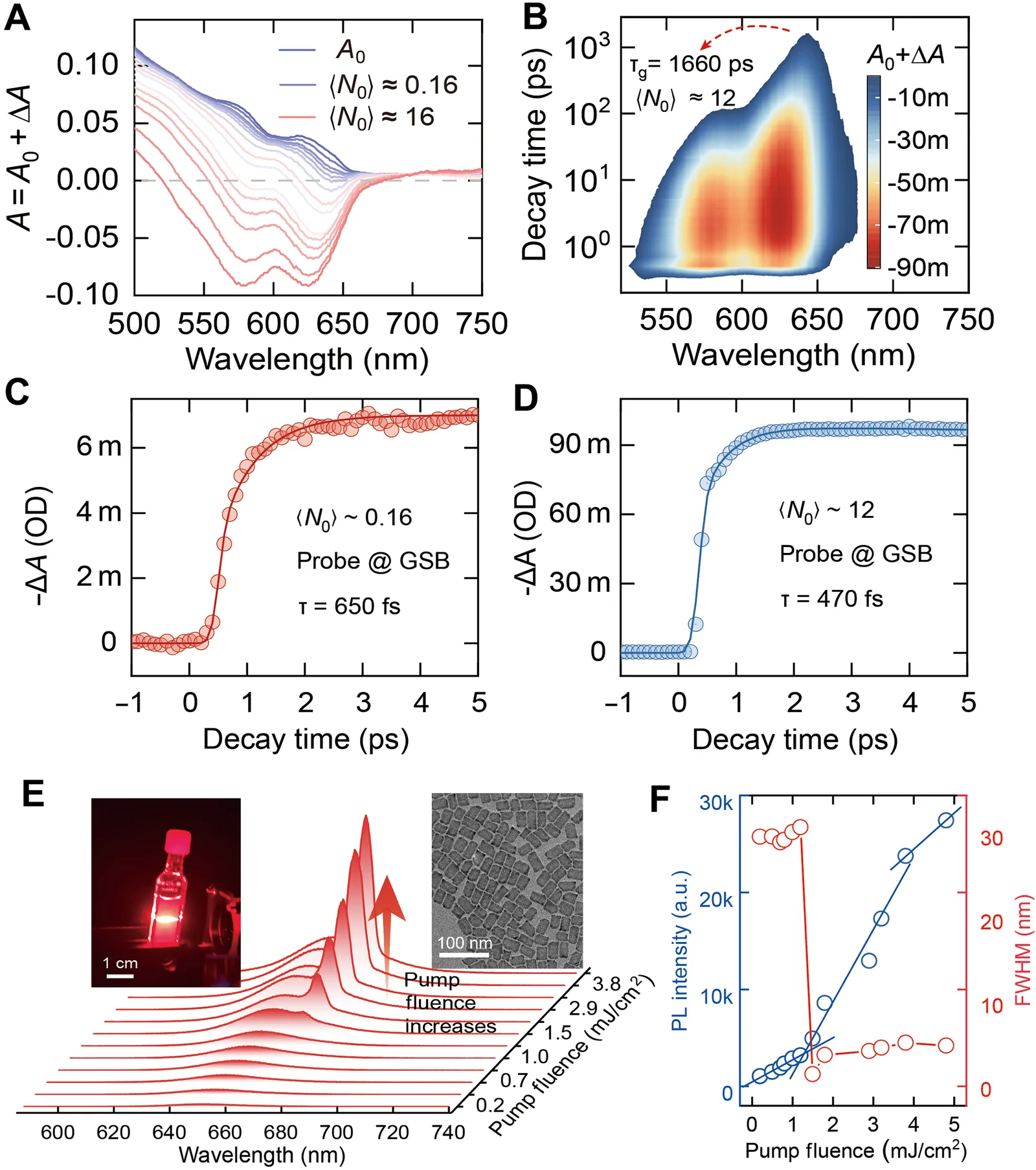
Optical properties of CdSe/CdSeS/CdZnS core/graded-crown/graded-shell CNPs. (**A**) Nonlinear absorption TA spectra (Δ*Α* + *A*_0_) obtained the TA spectra (Δ*Α*) of early delay time (2 ps) under various numbers of 〈*N*_0_〉 to the steady-state absorption spectrum (*Α*_0_). Below the dashed line, *A* < 0 indicates optical gain. (**B**) 2D fs-TA spectra of the gain spectra at 〈*N*_0_〉 ≈ 12 as functions of both wavelength and delay time. The gain lifetimes (τ_g_) are 1660 ps at 640 nm. (**C** and **D**) By fitting the GSB extracted from the fs-TA spectral evolution data on the early ps timescale, the HC cooling times for 〈*N*_0_〉 ∼0.16 (C) and 〈*N*_0_〉 ∼12 (D) were obtained. Excitation wavelength at 530 nm. (**E**) ASE measurement of liquid CdSe/CdSeS/CdZnS core/crown/shell CNPs under nanosecond pulse laser excitation. The insets show the fluorescence images of the CNPs samples. (**F**) Evolution of the normalized PL intensity and the FWHM of emission peaks as a function of different pump fluence for liquid CNPs ASE.

We used CdSe/CdSeS/CdZnS core/crown/shell CNPs as the gain medium (*29*, *30*), whose basic optical properties and structural characteristics are summarized in figs. S5 and S6. The heterostructure was constructed through a sequential lateral-growth and shell-growth strategy. First, a graded-alloy CdSeS “crown” was grown laterally on the edges of the CdSe nanoplatelets, which helps suppress ripening, dissolution, and structural deformation during the subsequent high-temperature shell-growth process. A uniform CdZnS alloy shell was then deposited to passivate surface defects and reduce nonradiative recombination, thereby improving the gain quality of the CNPs (Fig. 1E). Transmission electron microscopy analysis shows that the CdSe/CdSeS/CdZnS core/crown/shell CNPs have an average length ∼30 nm, a width of ∼20 nm, and a thickness of ∼5 nm (Fig. 2E and fig. S6). The CNPs exhibit a nearly rectangular morphology with well-defined edges, consistent with the anisotropic growth strategy described in Materials and Methods.

The ultrafast exciton dynamics and optical gain properties of CNPs are critical determinants of their lasing performance (*31*, *32*). In these materials, optical gain emerges from a competition among multiexciton dynamics, Auger-related processes, hot-carrier cooling, and recombination through local defect states (*33*). We investigated the ultrafast exciton dynamics of CNPs employing pump fluence–dependent fs–transient absorption (TA) spectroscopy. As the pump fluences (*j*_p_ in the unit of μJ cm^−2^) increases, the ground-state bleaching (GSB) signal of the CNP gradually saturates. Referring to a previous literature (*34*), the linear absorption cross section (σ_lin_) of the CNP at 530 nm is approximately 3.6 × 10^−15^ cm^2^ (see fig. S7). According to the 〈*N*_0_〉 = σ_lin_ × *j*_p_, we can estimate the initial average number of excitons per CNP. We subsequently employed TA to analyze optical gain and ascertain its gain lifetime. The early-time TA spectra (Δ*A*) with a delay of 2 ps are combined with the steady-state absorption spectrum (*A*_0_) to derive the nonlinear absorption spectra: *A*′ = Δ*A* + *A*_0_. As illustrated in Fig. 2A, *A*′ diminishes with rising 〈*N*_0_〉 and ultimately becomes negative in the long-wavelength domain, indicating optical gain. Figure S8A shows the decay dynamics monitored at the GSB feature (∼636 nm) for 〈*N*_0_〉 values ranging from 0.16 to 16. The kinetic traces are normalized and scaled according to the long-lived tail associated with the single-exciton state, allowing the excitation-density-dependent multiexciton dynamics to be compared more clearly. The inset schematically illustrates the biexciton optical-gain process. To further visualize the evolution of optical gain, we plot the time-dependent full gain spectra in Fig. 2B, which gradually decay as multiexciton recombination proceeds. By subtracting kinetic traces measured at different 〈*N*_0_〉, we isolate the fast decay component associated with biexciton recombination (fig. S8B). This component can be fitted with a single-exponential decay, yielding a biexciton lifetime (τ_XX_) of 700 ps (*35*). This lifetime is substantially longer than the typical biexciton lifetimes of conventional CQDs, which are usually on the order of tens of picoseconds (*36*, *37*), indicating strongly suppressed Auger recombination and thereby favoring the establishment of biexciton-mediated population inversion.

Specifically, we determine optical gain lifetimes (τ_g_) of 121 and 1660 ps at 585 and 640 nm, respectively, achieving optical gain (with a gain width of 467 meV) over a wide spectral range from 535 to 670 nm. The two gain bands at 585 and 640 nm originate from the 1*S*_lh_-1*S*_e_ and 1*S*_hh_-1*S*_e_ transitions, respectively. Fast intraband scattering between valence sub-bands, together with multiexciton Auger processes, drives holes from the higher-energy light-hole (LH) sub-band into the heavy-hole (HH) sub-band (*38*, *39*). As a result, population inversion of the LH transition at 585 nm can only be maintained when the number of excitons is relatively large in the early stages (*40*). Meanwhile, the CNPs exhibit stronger linear and excited-state absorption at 585 nm, which means that when the multiexcitons are slightly depleted, their net gain is quickly offset by reabsorption (*41*). In contrast, the gain at 640 nm is mainly contributed by the biexciton/multiexciton stimulated emission of the lowest 1*S*_hh_-1*S*_e_ state and the biexciton lifetime studied previously was 700 ps, which also contributed to the increase in optic gain lifetime. The band-edge gain lifetime of 1660 ps at 640 nm observed here exceeds previously reported values for core-shell quantum dots (*42*–*44*). Within the optical gain band, the relaxation dynamics of carriers are governed not only by Auger recombination but also by hot-carrier cooling (*45*, *46*). By fitting the early-time evolution of the GSB (Fig. 2, C and D), we extract hot-carrier cooling times of ∼650 fs at low excitation (〈*N*_0_〉 ≈ 0.16) and ∼470 fs at high excitation (〈*N*_0_〉 ≈ 12). At high excitation densities, multiexciton relaxation, Auger-mediated energy exchange, and band-edge stimulated emission effectively accelerate the hot carriers cooling (*33*). Simultaneously, the presence of HH and LH sub-bands provides more in-band scattering channels, thus suppressing the thermal phonon bottleneck effect. This facilitates the rapid transfer of high-energy multi-excitons to the edge of the HH band, which is more conducive to the formation of population inversion.

The amplified spontaneous emission (ASE) behavior of colloidal CNP solutions was studied using nanosecond pulsed laser excitation. As shown in Fig. 2E, at low pump fluence, only broad spontaneous photoluminescence (PL) was observed. As the pump fluence increased, a distinct narrow spectral peak emerged on the red side of the PL spectrum, with a full width at half maximum of about 5 nm, indicating the onset of ASE. Furthermore, the ASE intensity increased rapidly with increasing pump fluence. By analyzing the superlinear dependence of the PL intensity on pump fluence, we determined the ASE threshold to be as low as 1.3 mJ·cm^−2^ (Fig. 2F), which, to the best of our knowledge, is among the lowest reported for colloidal nanocrystal gain media. The inset shows the fluorescence image of the CNP sample under nanosecond pumping. Because of ASE-enhanced light scattering, the entire cuvette emitted strong red light. Notably, a bright ASE emission line was clearly visible at the outer edge of the pump region, further confirming the occurrence of ASE and its directional enhancement.

These results demonstrate that the prolonged biexciton lifetime and suppressed Auger recombination in our CNPs effectively facilitate population inversion, enabling low-threshold optical amplification, a key prerequisite for high-performance liquid-phase lasing. However, the realization of a stable, high-coherence laser output depends not only on the intrinsic gain properties of the material but also on the dynamic behavior of the colloidal nanoparticles within the liquid environment, which can profoundly affect gain uniformity, cavity stability, and overall lasing performance.

### Self-organized optofluidic locking of anisotropic CNPs

Although the CdSe/CdSeS/CdZnS core/crown/shell CNPs developed here exhibit sufficiently long gain lifetime and low-threshold optical amplification, these material advantages alone are not enough to guarantee stable lasing in a liquid environment. As discussed above, the realization of a stable high-coherence output depends not only on the intrinsic gain properties of the material, but also on the dynamic behavior of the colloidal nanoparticles within the liquid cavity. In our liquid-state system, we found that the output instability did not arise from a single factor, but from the coupled evolution of particle migration, local heat accumulation, and refractive-index fluctuation. Under optical excitation, the spatial distribution of CNPs can be continuously perturbed, while the accompanying thermal gradients further destabilize the intracavity field. These observations led us to reconsider the gain medium not as a passive emitter embedded in a fluctuating liquid, but as an active component whose light-driven response might be harnessed to counteract the very instability it experiences.

Self-organization of nanoparticles under external fields has been widely explored as a route to directed assembly, where interparticle interactions and externally applied forces jointly guide colloidal systems toward ordered or functional configurations (*47*–*50*). Conventional optical tweezers, by contrast, typically use tightly focused optical fields to trap and manipulate isolated dielectric particles through optical gradient and scattering forces (*51*–*53*), and have also been extended to anisotropic nanostructures for optical manipulation, trapping, binding, and rotational control (*54*, *55*). Our optofluidic locking process differs from both static field–directed assembly and single-particle trapping: The auxiliary continuous-wave optical field induces a dynamically maintained redistribution of anisotropic CNPs within an operating liquid gain medium, where optical forces, interparticle interactions, and thermally driven convection collectively drive CNP migration, orientational ordering, and accumulation near the optically favored region. On the basis of this idea, we developed the concept of optofluidic locking for the CNP liquid laser.

**Fig. 3.**
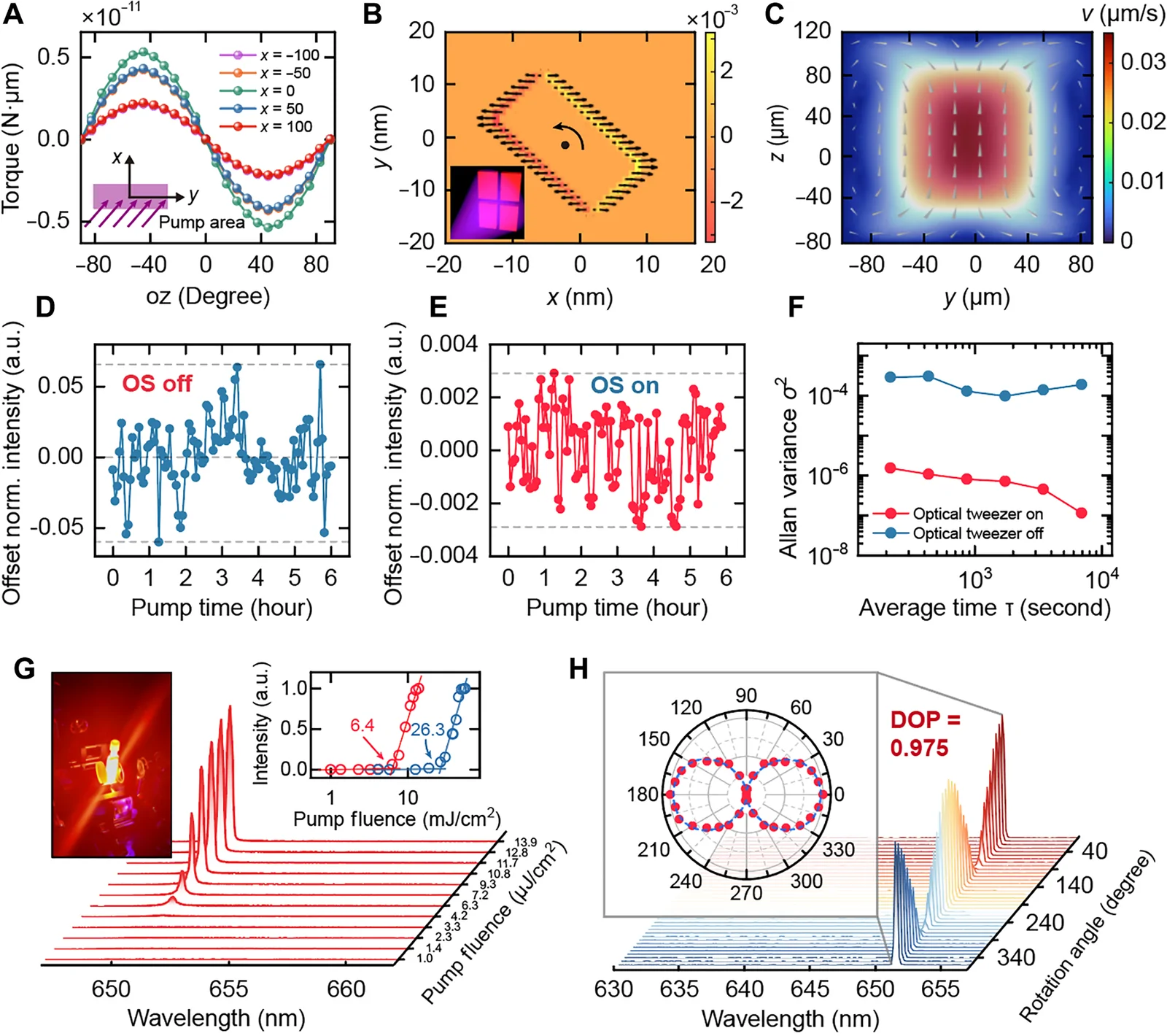
Optofluidic locking and its impact on lasing stability. (**A**) Calculated optical torque acting on CNPs as a function of position along the *x* axis, with the Gaussian-beam center defined as *x* = 0. (**B**) Optical-gradient force distribution acting on CNPs at a rotation angle of *oz* = 45°; the color scale and arrow length indicate the force direction and magnitude, respectively. (**C**) Simulated laminar-flow field of the CNP fluid in the *y*-*z* plane, showing thermally driven convection (coordinate axes are defined in fig. S15). (**D**) Laser output intensity versus pump time without the auxiliary optical locking field (unstabilized condition). (**E**) Laser output intensity versus pumping time with the auxiliary optical locking field enabled, demonstrating optofluidic self-stabilization. (**F**) Allan variance of the output intensity with and without the auxiliary optical locking field. (**G**) Evolution of the laser spectrum with increasing pump fluence for the optofluidically self-stabilized CNP liquid laser. Inset: lasing thresholds with the auxiliary optical locking field enabled and disabled. (**H**) Normalized laser intensity as a function of polarization angle. Inset: corresponding intensity scatter plot and fitted curve.

To test whether such a self-organized state could indeed emerge, we carried out numerical simulations to track the optomechanical evolution of CNPs under continuous-wave illumination. The results reveal that the particles undergo coordinated collective motion driven by the subtle interplay between optical gradient and scattering forces. When the CNPs are horizontally aligned (*oz* = 0°; Fig. 3A), the net optical torque approaches zero, and the system resides in a near-equilibrium state. However, when the assembly is tilted to *oz* = 45° (Fig. 3B), this symmetry is broken, creating an imbalance in the optical pressure distribution and generating a finite restoring torque. The resulting torque initiates rotational motion and drives the system back toward its preferred horizontal orientation. Through repeated self-correcting cycles of displacement and reorientation, the CNPs gradually migrate toward the region of highest optical intensity and accumulate near the beam center. In this dynamically maintained state, the optical torque aligns the long axes of the CNPs approximately perpendicular to the direction of light propagation. Meanwhile, weak field-mediated optical-binding interactions promote the formation of loosely spaced chain-like assemblies rather than compact stacking. Owing to the weak nature of these interactions, the CNPs retain angular deviations of several degrees in the final quasi-equilibrium state (fig. S14). We refer to this collective behavior as optical “locking.” Distinct from the optical trapping of isolated particles, the defining feature of this process is the emergence of a dynamically maintained collective state that continuously reshapes the gain landscape into a more stable lasing configuration.

Beyond the direct action of optical forces, fluid dynamics also plays a crucial role in establishing the stabilized lasing state. To obtain a more complete physical picture, we further performed coupled multiphysics simulations incorporating laminar flow and heat transfer. As shown in Fig. 3C, a gentle upward flow develops near the bottom of the cuvette in the *y*-*z* plane, driven by the combined action of gravity and thermally induced buoyancy. This convective flow acts as a natural cooling channel, efficiently dissipating heat, suppressing local hot spots, and flattening the temperature gradient across the gain region. As a result, thermal degradation is reduced and the spatial and spectral stability of the cavity field is substantially improved. The stabilized laser state therefore arises not from optical locking alone, but from the cooperative interplay of optical, thermal, and fluidic effects, which together establish a dynamically self-regulated lasing environment.

To place the optical and thermally induced fluidic effects on a common mechanical scale, we further estimated the hydrodynamic drag acting on the CNPs based on the simulated convective flow field (fig. S17). The maximum convection-induced Stokes drag acting on the CNPs is comparable in magnitude to the stronger *y*-direction component of the optical-gradient force, with both being approximately 10^−16^ N W^−1^. Optical forces primarily provide orientational locking of the CNPs. In contrast, thermally induced flow mainly reduces local heat accumulation and refractive-index gradients. This self-regulating mechanism has a direct and pronounced impact on the lasing characteristics. As shown in Fig. 3G, when optofluidic locking is enabled, the lasing threshold decreases markedly from 26.3 to 6.4 mJ cm^−2^, corresponding to a reduction of more than 75%. Such a substantial decrease indicates that optical locking and convective cooling act synergistically to maintain a more favorable gain distribution and to lower the effective excitation requirement for laser oscillation. This result not only supports the physical validity of the optofluidic locking concept but also highlights its practical value for reducing the energy cost of liquid-state lasing.

We next monitored the temporal evolution of the normalized output intensity to evaluate the long-term stability of the liquid laser. Without optofluidic stabilization, the laser output exhibited pronounced fluctuations with an amplitude exceeding 12% (Fig. 3D). Once the auxiliary beam was applied and optofluidic locking was activated, these fluctuations were markedly suppressed to approximately 0.6%, and the system maintained stable operation for as long as 6 hours (Fig. 3E). To further quantify stability over different averaging times, we calculated the Allan variance of the output signal (see Materials and Methods). As shown in Fig. 3F, when the auxiliary optical locking field was turned off, the Allan variance remained relatively high, reaching its minimum value of 9.67 × 10^−5^ at an averaging time of 1.7 × 10^3^ s. In contrast, with the auxiliary optical locking field enabled, the laser exhibited substantially improved long-term stability, achieving a minimum Allan variance of 1.14 × 10^−7^ at 6.9 × 10^3^ s. These results demonstrate an approximately three-orders-of-magnitude enhancement in signal stability and confirm that optofluidic self-stabilization effectively suppresses both rapid output noise and long-timescale drift. We further examined whether the high-density optofluidically locked region could evolve into irreversible aggregation or sedimentation during extended operation. The CNPs were continuously locked by the CW optical field for 72 hours. No obvious precipitation was observed within this test window. Meanwhile, the CNP laser intensity was recorded every 6 hours. As shown in fig. S18, the results indicate that the system exhibited no obvious monotonic intensity decay or long-term degradation within 72 hours.

The collective orientational locking of CNPs is also reflected in the polarization behavior of the output laser. Figure 3H shows the measured normalized laser intensity as a function of polarization angle together with the fitted curve. Fitting based on Malus’ law yields a degree of polarization (DOP) exceeding 97.5% ± 0.3%, indicating a highly ordered polarization state. This strong polarization originates from the quasi-two-dimensional geometry and anisotropic optical response of the CNPs. When the CNPs are randomly oriented in the liquid phase, the transition dipoles of different particles are distributed over different directions, causing the emission from different polarization components to be partially averaged and preventing the formation of a highly polarized output. Upon application of the auxiliary continuous-wave optical field, optical torque drives the anisotropic CNPs to rotate toward preferred orientations, thereby narrowing their orientational distribution and establishing a dynamically maintained, collectively ordered state. Because the band-edge transition dipoles of CNPs predominantly lie within the nanoplatelet plane, this orientational ordering introduces a polarization-dependent optical response and gain anisotropy. Repeated cavity feedback and polarization-mode competition can further enhance this anisotropy, favoring laser oscillation along a dominant polarization direction. To distinguish the general optofluidic stabilization effect from the anisotropy-specific orientational-locking effect, we performed a control experiment using near-spherical CQDs as an approximately isotropic gain medium under otherwise identical conditions. The corresponding results, presented in figs. S19 to S21, reveal the differences in polarization behavior and further underscore the unique role of CNP anisotropy in achieving the high DOP observed in our system.

Having established that optofluidic locking can simultaneously lower the lasing threshold, suppress output fluctuations, enhance long-term Allan-variance stability, and impose strong polarization ordering, we next ask whether these gains in stability can be preserved while extending the functionality of the laser along another important axis: wavelength agility. In practical applications, high spectral purity is most valuable when combined with broad and controllable tunability.

### Wavelength-agile single-frequency lasing with preserved spectral purity

**Fig. 4.**
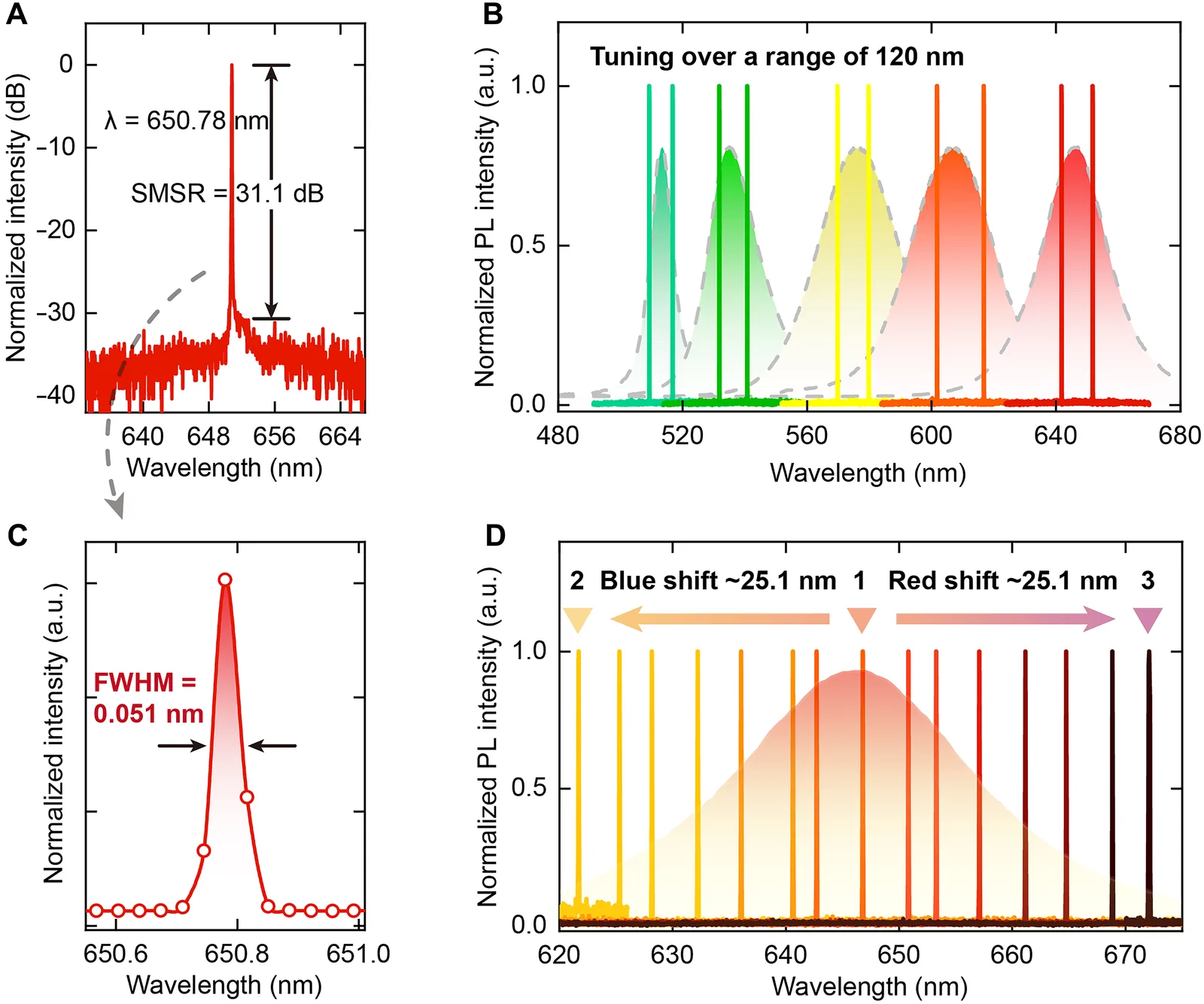
Spectral characteristics and wavelength tunability of the CNP liquid laser. (**A**) Side-mode suppression ratio (SMSR) of the CNP liquid laser. (**B**) Linewidth of the single-frequency lasing emission. (**C**) Wavelength tunability of the CNP liquid laser, demonstrating single-frequency lasing in five distinct spectral bands. (**D**) Continuous wavelength tunability of the liquid-phase laser using CdSe/CdSeS/CdZnS core/crown/shell CNPs as the gain medium, with a total tuning range exceeding 50 nm.

To investigate wavelength agility, we combined the optofluidically stabilized external cavity with band-engineered CNP gain media. Benefiting from the stabilized gain distribution established through optofluidic locking, the device maintained high spectral purity throughout the tuning process, exhibiting a SMSR above 31 dB and an ultranarrow single-frequency linewidth of 0.051 nm, corresponding to a quality factor exceeding 12,500 (Fig. 4, A and B). These results show that the spectral advantages enabled by optofluidic locking can be preserved under active wavelength tuning, rather than being limited to a single operating point.

We then expanded the accessible tuning window through a materials-level design strategy. By varying the ligand environment, heterostructure configuration, and nanoplatelet thickness, we synthesized and optimized five distinct CNP gain media, including CdSe/CdSeS/CdZnS core/graded-crown/graded-shell CNPs, orange-emitting CdSe/CdZnS core/thin-shell CNPs, yellow-emitting CdSeS/CdZnS core/shell CNPs, green-emitting bromide-ligand-capped CdSe CNPs, and CdSe/CdSeS core/alloyed-crown CNPs. By combining the optofluidically stabilized external cavity with band-engineered CNP gain media, the present liquid-laser platform achieves single-frequency spectral coverage from ∼500 to 672 nm, corresponding to a total accessible range of more than 170 nm (Fig. 4C). For an individual CdSe/CdSeS/CdZnS core/crown/shell CNP gain medium, continuous cavity tuning around the central lasing wavelength of ∼622.6 nm further provides a 25.1 nm blue shift and a 25.2 nm red shift, corresponding to a total continuous tuning range of approximately 50.3 nm (Fig. 4D; see also section S7).

To assess the practical competitiveness of the present liquid laser, we compared its spectral purity with that of representative commercial laser diodes. As summarized in table S1, the SMSR of our device exceeds 31 dB, placing it in the spectral-purity regime of representative single-frequency diode sources. This comparison is further visualized in table S1, where the emission linewidth of our system is benchmarked against representative commercial models. Together, these results show that the present CNP-based liquid laser approaches the performance regime of established solid-state coherent sources in terms of spectral purity, while retaining the distinctive advantages of liquid-phase tunability and reconfigurable gain-media design.

### Application-oriented pumping with a spectrally robust CNP liquid laser

**Fig. 5.**
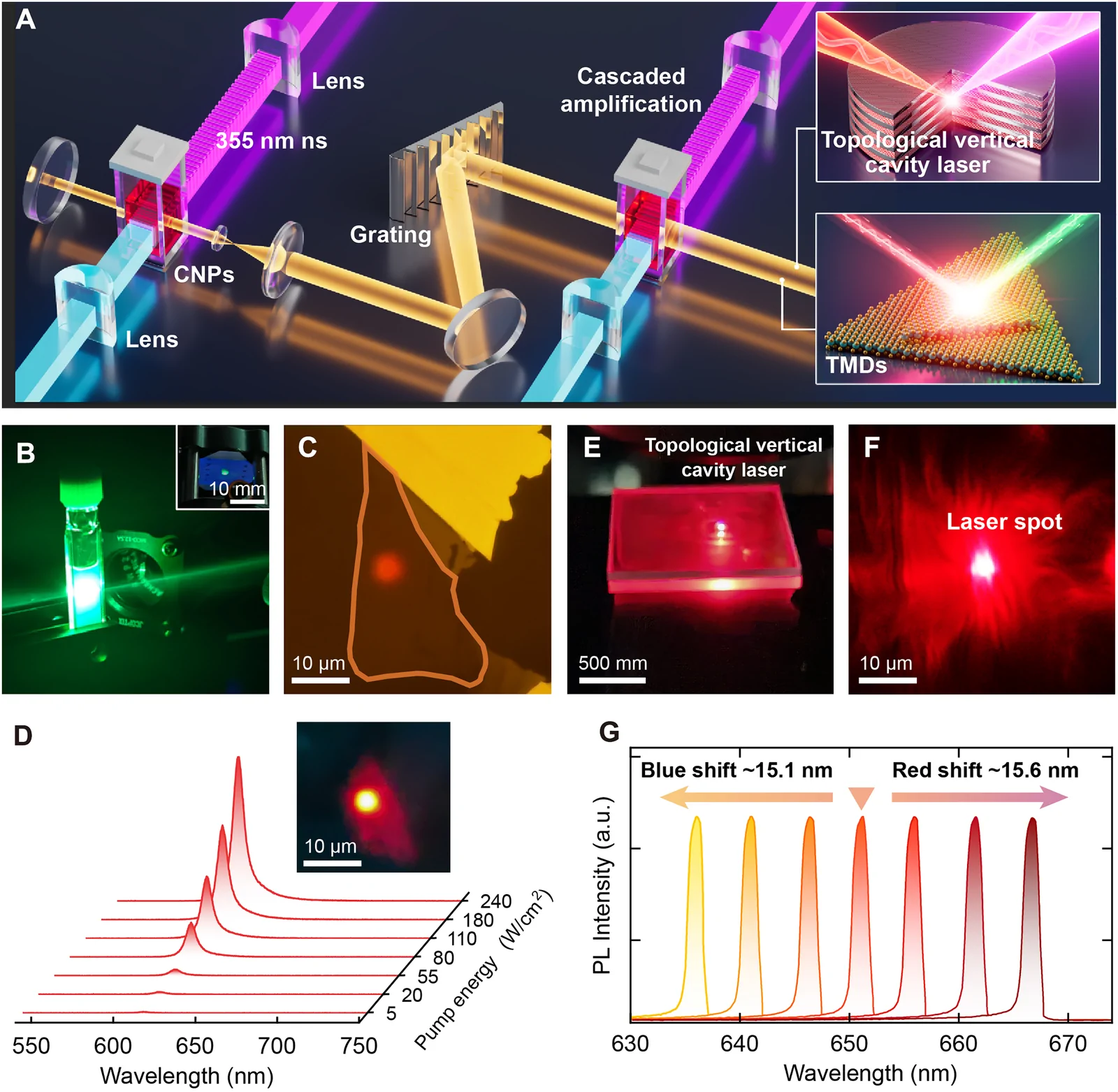
Application demonstrations of the CNP liquid laser. (**A**) Schematic of the cascaded amplification architecture used to pump monolayer WS_2_ and a topological vertical-cavity laser. (**B**) Dark-field image of the excitation setup, showing bright CNP luminescence from the cuvette; inset: output laser spot of the cascaded system. (**C**) Bright-field optical micrograph of a monolayer WS_2_ flake, with the monolayer region outlined in yellow. (**D**) PL from monolayer WS_2_ pumped by the CNP liquid laser; inset: PL image of the excitation region. (**E**) Photograph of the topological vertical-cavity laser device. (**F**) Dark-field image of the excitation spot on the topological vertical-cavity device. (**G**) Continuous wavelength tuning of the topological vertical-cavity laser under pumping by the CNP liquid laser.

Having shown that the present liquid laser can deliver diode-grade spectral purity together with broad wavelength agility, we next asked whether these capabilities could be translated into a more demanding functional role: serving as a practical pump source for downstream photonic systems. This is a critical test of the platform, because spectral purity and tunability alone are not sufficient for real utility unless they can be maintained at an output level compatible with external-device excitation. We therefore moved beyond intrinsic laser metrics and examined whether the self-stabilized CNP laser could operate as an efficient and spectrally robust excitation source under application-relevant conditions. To meet the power requirement for such experiments, we implemented a cascaded amplification architecture (Fig. 5A), which increased the output power to ∼4.5 mW while preserving the spectral consistency of the laser emission, corresponding to an enhancement of ∼500% relative to a single-stage configuration. The dark-field image of the excitation setup (Fig. 5B) further confirms stable laser generation, as evidenced by the bright output spot and the characteristic green luminescence from the CNP-filled cuvette.

We first used the amplified liquid laser to excite PL from monolayer WS_2_ (Fig. 5C). A pronounced emission peak emerged at ∼614 nm with a full width at half maximum of ∼7.9 nm (Fig. 5D), consistent with radiative recombination of the A exciton associated with the direct bandgap of monolayer WS_2_. The narrow linewidth and monotonic increase in PL intensity with pump fluence indicate that the CNP laser provides efficient and well-controlled excitation for two-dimensional semiconductors. We then turned to a more cavity-sensitive test case, namely, a topological vertical-cavity laser (Fig. 5, E and F, and fig. S23). Building on our previous demonstration that topological interface states can strengthen field localization and support continuous-wave optical pumping (*6*), we used the CNP liquid laser to drive the topological cavity while tuning the thickness of the CNP layer through self-assembly. Under pumping by a single CdSe/CdSeS/CdZnS core/crown/shell CNP liquid laser, the topological vertical-cavity device exhibited continuous wavelength tuning from 636.1 to 666.8 nm, corresponding to a tuning span of 30.7 nm (Fig. 5G). Together, these application-oriented demonstrations show that the present liquid-laser platform is not only spectrally pure and tunable as a source in itself, but can also function as a stable and versatile pump for advanced photonic and optoelectronic systems.

## DISCUSSION

In summary, by integrating high-performance CNP gain media with optofluidic locking in a Littman-like external cavity, we establish a self-stabilizing strategy for achieving high-coherence single-frequency lasing in dynamic liquid gain media. Rather than treating fluidic fluctuations solely as a source of instability, this approach harnesses the coupled action of optical forces and convective flow to reshape the gain distribution and stabilize laser operation. Leveraging the ultralong gain lifetime and stabilized inversion dynamics enabled by the CdSe/CdSeS/CdZnS core/graded-crown/graded-shell architecture, the system maintains exceptional spectral purity and robust single-mode operation. The optofluidic self-stabilization mechanism, combining optical locking and convective cooling, suppresses fluctuations and system noise, delivering a SMSR above 31 dB together with an ultranarrow linewidth of 0.051 nm (*Q* > 12,500), thereby accessing a high-spectral-purity regime for colloidal liquid lasers. Notably, optofluidic self-stabilization drives the output stability to an Allan-variance floor of 1.14 × 10^−7^, corresponding to an approximately three-orders-of-magnitude enhancement in signal stability. Simultaneously, the platform enables single-mode spectral coverage spanning 170 nm across band-engineered CNP gain media and sustains hour-long stable operation. Together, these results identify optofluidic locking as a general route for stabilizing liquid-state lasers and advancing colloidal gain media toward practical high-coherence photonic sources.

**Fig. 6.**
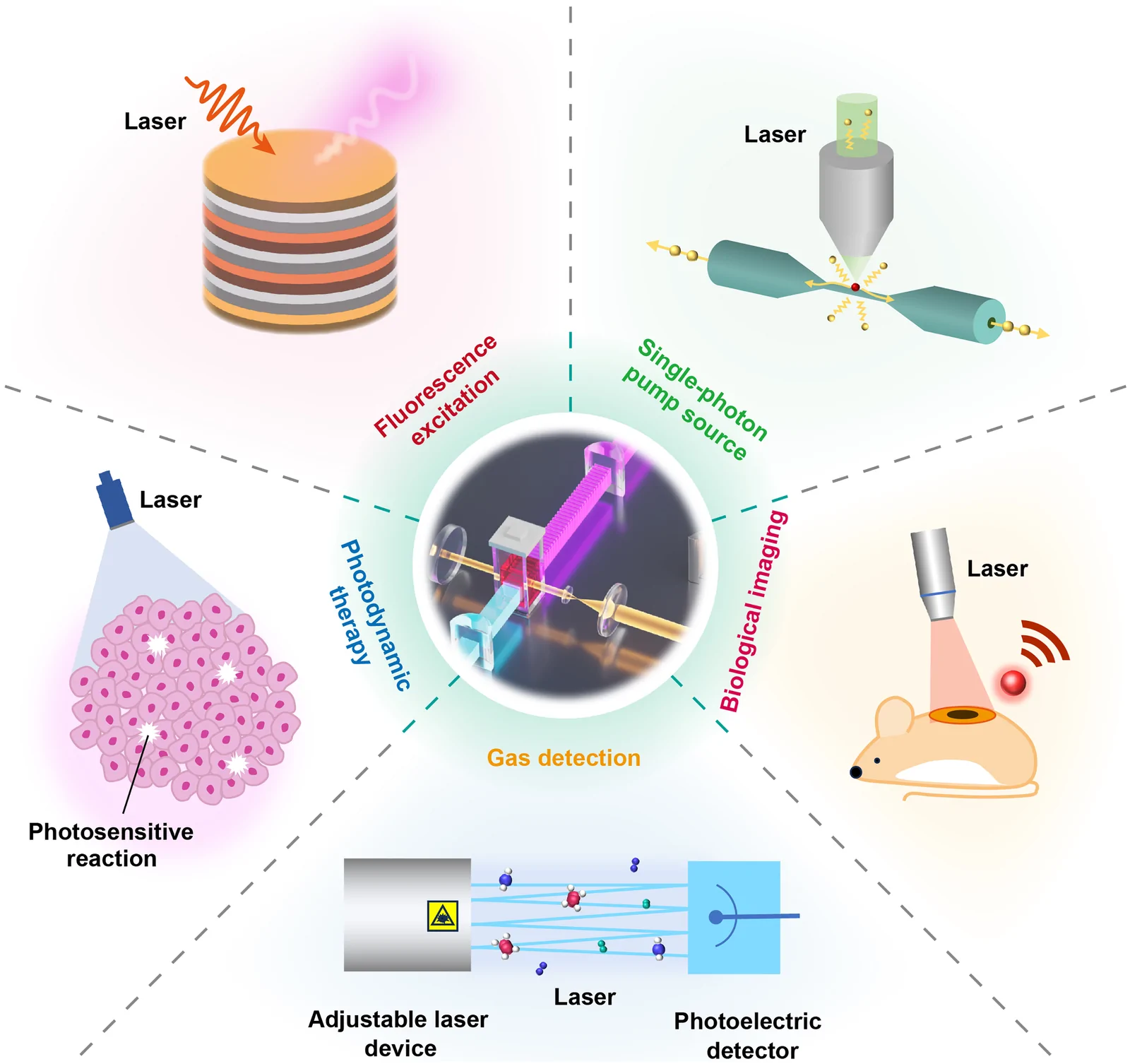
Broad applicability and universality of the CNP liquid laser. The proposed liquid-laser platform provides a general route for investigating fundamental photophysical phenomena and enabling diverse applications, including the pumping of single-photon emitters, fluorescence excitation, gas sensing, photodynamic therapy, and bioimaging.

Looking forward, the versatility of our CNP liquid-laser platform provides a framework for translating dynamically reconfigurable liquid gain media into deployable coherent-light technologies (Fig. 6). Its high spectral purity, ultranarrow linewidth, wide spectral coverage, and long-term stability make it a compelling pump engine for quantum photonics, where narrow linewidth and precise wavelength control are essential for addressing single-photon emitters and other quantum systems. In bioimaging and fluorescence excitation, a spectrally pure and tunable source can improve contrast, reduce background contributions, and facilitate multiplexed or hyperspectral readout. The narrow emission line further enables accurate matching to molecular absorption features, supporting high-resolution spectroscopy and gas sensing with enhanced selectivity. In addition, visible-band wavelength agility combined with liquid-phase thermal management offers opportunities for photodynamic therapy, where wavelength-matched activation of photosensitizers is desired. More broadly, the integration of external-cavity control with optofluidic self-stabilization points toward miniaturized and integrated liquid-laser architectures, including microfluidic and chip-scale implementations, and suggests a possible pathway toward electrically driven liquid-state coherent sources. These advances extend the relevance of colloidal liquid lasers beyond proof-of-concept demonstrations and toward real-world photonic systems that require both spectral precision and dynamic reconfigurability.

## MATERIALS AND METHODS

### Synthesis of CdSe/CdSeS/CdZnS core/crown/shell CNPs

CdSe/CdSeS/CdZnS core/crown/shell CNPs were synthesized as follows: 1 ml of Cd_0.25_Zn_0.75_OA_2_ (0.2 M), 9 ml of ODE, and 200 μl of OA were placed in a 50-ml three-necked flask. The mixture was degassed at 100°C for 30 min and cooled to 80°C. At this temperature, 1 ml of purified CdSe/CdSeS CNPs in hexane (OD > 90 at the first excitonic peak) was injected, followed by degassing for 10 min. Then, 1 ml of OAm was injected, and the temperature was raised to 300°C under nitrogen at a rate of 15°C/min. From 165° to 240°C, a solution of OTL-ODE was injected at 2.5 to 12.5 ml/hour (the injection rate can be varied to tune the emission wavelength); after reaching 240°C, the injection rate was adjusted to 5 ml/hour. The reaction was kept at 300°C for 1 hour and then cooled to room temperature with an air gun. Last, the product was purified several times with ethanol and hexane.

### Preparation of tunable single-frequency lasing from CNPs

As show in fig. S2, the experimental system is based on a CNP external-cavity laser using a modified Littman configuration. CNP dispersed in cyclohexane were used as the laser gain medium and loaded into a standard quartz cuvette with a width of 1 cm and an optical path length of 1 mm. Optical pumping was provided by a nanosecond Nd:YAG laser operating at 355 nm with a pulse duration of 7 ns and a repetition rate of 10 Hz. The pump beam was shaped into a stripe profile and focused along the width of the cuvette to achieve a uniform excitation region. Emission from the excited CNP solution was efficiently coupled into the external resonant cavity. A 442-nm continuous-wave laser with an output power of approximately 50 mW at the sample position was introduced as an auxiliary stabilization beam to generate optical gradient forces within the gain medium and enable optofluidic locking. After being focused by a cylindrical lens into the CNP solution, this auxiliary beam, in combination with thermally induced solvent convection, enabled optofluidic self-stabilization. The emission spectrum of the laser was measured using a high-precision spectrometer with a spectral resolution of 0.022 nm, and its linewidth, SMSR, and wavelength tuning range were determined.

### Allan variance analysis

Allan-variance analysis confirms that the auxiliary optical locking field significantly enhance the long-term stability of the laser intensity. The Allan variance is defined as (*56*)

$$\sigma^{2}\left(\tau \right)=\frac{1}{2\left(M-1\right)}\sum_{k=1}^{M-1}{\left(\bar{y_{k+1}}-\bar{y_{k}}\right)}^{2}$$

where τ is the averaging (integration) time, *M* is the number of laser intensity measurements, and $\bar{y_{k}}$ represents the mean deviation of the laser output intensity over a time interval of duration τ.

### Steady-state PL, absorption, and PL quantum yield measurements

Steady-state PL measurements were carried out using a custom-designed optical setup. A helium-cadmium (He-Cd) laser with an excitation wavelength of 442 nm served as the light source. The emitted signals were collected and analyzed using a monochromator coupled to a photomultiplier tube, with signal detection performed via a standard lock-in amplification technique. Absorption spectra were recorded using a Shimadzu RF-5301 PC spectrofluorometer with a spectral resolution of 0.5 nm. The PL quantum yield was measured using an Otsuka Quantum Efficiency Measurement System (QE2000), which incorporates an integrating sphere and a monochromator integrated with a 150-W xenon lamp, enabling accurate quantum yield determination.

### TA measurements

During the experiment, the 800-nm output of a Ti:sapphire regenerative amplifier (Spectra-Physics; 1 kHz repetition rate, ∼100 fs pulse duration) was split into two beams. One beam was routed through a mechanical delay stage and focused into a CaF_2_ crystal to generate a white-light supercontinuum, which served as the probe pulse (∼100 fs). The other beam was sent to a TOPAS optical parametric amplifier to produce the pump pulse (∼100 fs), which was modulated by an optical chopper operating at 500 Hz.

## Supplementary Materials

### This PDF file includes:

Supplementary Text Fig. S1 to S23 Tables S1 to S4 References
