# Supplementary material for: Self-organized optofluidic locking enables stable single-frequency lasing in dynamic liquid media
Source: Sci Adv. 2026 Sep 23;12(39):eaei3045. doi: 10.1126/sciadv.aei3045 (PMC13600248; doi:10.1126/sciadv.aei3045)
Supplement: Supplementary file 1 [file sciadv.aei3045_sm.pdf]

Science Advances
AAAS

Supplementary Materials for

Self-organized optofluidic locking enables stable single-frequency lasing in dynamic liquid media

Rui Duan *et al.*

Corresponding author: Junzi Li, jzli@szu.edu.cn; Yichen He, heyichen_9112@tju.edu.cn; Jianqi Qi, qijianqi@scu.edu.cn; Huiyun Liu, huiyun.liu@ucl.ac.uk; Handong Sun, hdsun@um.edu.mo

Sci. Adv. **12**, eaei3045 (2026)
DOI: 10.1126/sciadv.aei3045

This PDF file includes:

Supplementary Text
Fig. S1 to S23
Tables S1 to S4
References

S1. Chemicals.

Sodium myristate (Na(myr), Macklin >98%), cadmium nitrate tetrahydrate ($Cd(NO_3)_2 \cdot 4H_2O$, Sigma-Aldrich 98%), cadmium acetate dihydrate ($Cd(ace)_2 \cdot 2H_2O$, Sigma-Aldrich 98%), cadmium acetate ($Cd(ace)_2$, Sigma-Aldrich 99.99%), zinc acetate ($Zn(ace)_2$, Sigma-Aldrich 99.99%), selenium powder (Se, Sigma-Aldrich 99.99%), sulfur powder (S, Thermo Fisher Scientific >99.5%), 1-octadecene (ODE, Sigma-Aldrich 90%), 1-octanethiol (OTL, Sigma-Aldrich 98%), oleic acid (OA, Sigma-Aldrich 90%), oleylamine (OAm, Sigma-Aldrich 70%), hexane (Macklin HPLC), methanol (Macklin HPLC), ethanol (Macklin HPLC).

S2. Precursor preparation

S2.1. Cadmium myristate ($Cd(myr)_2$): The initial stage of the synthesis process entails the dissolution of 1.85 g of $Cd(NO_3)_2 \cdot 4H_2O$ in 60 mL of methanol. Concurrently, 4.7 g of Na(myr) should be dissolved in 375 mL of methanol. Subsequently, the two solutions should be combined, stirred, and filtered. Following a thorough cleansing of the mixture on five separate occasions and the substance is dried under a nitrogen atmosphere for 12 h.

S2.2. S suspension with OA (S-Sus-OA): The method of preparation for S-Sus-OA is outlined as follows: 32 mg of sulfur powder is to be dissolved in 10 mL of ODE and 330 μL of OA, following which the mixture is to be sonicated for a period of 15 min.

S2.3. Mixture of cadmium oleate and zinc oleate ($Cd_{0.25}Zn_{0.75}OA_2$): The following reagents are to be combined in a three-neck flask: 230.5 mg of $Cd(ace)_2$ (1 mmol), 550.4 mg of $Zn(ace)_2$ (3 mmol), 17 mL of ODE and 3 mL of OA. The mixture was degassed at 100°C for 60 min, after which it was heated to 240°C under nitrogen atmosphere. Following the resolution of the mixture, the solution was cooled to ambient temperature and stored within a nitrogen cabinet.

S2.4. OTL in ODE (OTL-ODE): A quantity of 42 μL OTL (0.24 mmol) was mixed with 5 mL ODE in an inert nitrogen atmosphere and stirred for a period of 60 min.

S3. Synthesis of CdSe/CdSeS Core/Crown CNPs

All reagents were employed without additional purification. The synthesis of CdSe/CdSeS core/crown nanoplatelets was performed as follows:

340 mg of Cd(myr)$_2$, 24 mg of selenium powder, and 30 mL of ODE were added into a three-necked flask. The mixture was degassed at room temperature for 30 min and then at 100 °C for 10 min. Under nitrogen atmosphere, the temperature was raised to 240 °C at a heating rate of 10–12 °C/min. When the temperature reached 200–205 °C, 120 mg of Cd(ace)$_2$·2H$_2$O was introduced. Maintaining the reaction process for 6 min after introducing the Cd(ace)$_2$·2H$_2$O, S-Sus-OA was injected at a rate of 18 mL/h for 5 min. The reaction was then cooled to 160 °C using an air gun, and 0.6 mL of OA was injected. Degassing was carried out at 110 °C for 10 min to remove H_2S. Below 70 °C, 2 mL of OA and 5 mL of hexane were added. The product was purified several times with ethanol and hexane.

S4. Transmission Electron Microscopy (TEM) Characterization

Elemental distribution was analyzed by energy-dispersive spectroscopy (EDS) mapping using the JEOL JSM-7800F PRIME. TEM characterization of the core/shell NPLs was conducted with a JEOL JEM 1400 transmission electron microscope operated at 100 kV. Colloidal NPL samples were drop-cast onto carbon-coated copper grids with a diameter of 3.05 mm for TEM observation.

S5. Measurement of Amplified Spontaneous Emission (ASE) Spectra

ASE spectra were measured using both a nanosecond pulsed laser (wavelength: 355 nm, pulse duration: 7 ns, repetition rate: 10 Hz). Edge emission as a function of pump intensity was collected using a fiber-coupled spectrometer and detected with a silicon charge-coupled device (CCD). The excitation spot size was recorded using a Thorlabs DCC1645C USB 2.0 CMOS microscopy camera, and the corresponding spot area was subsequently determined to calculate the pump fluence from the measured pump intensity.

S6. Comparison of Laser Performance

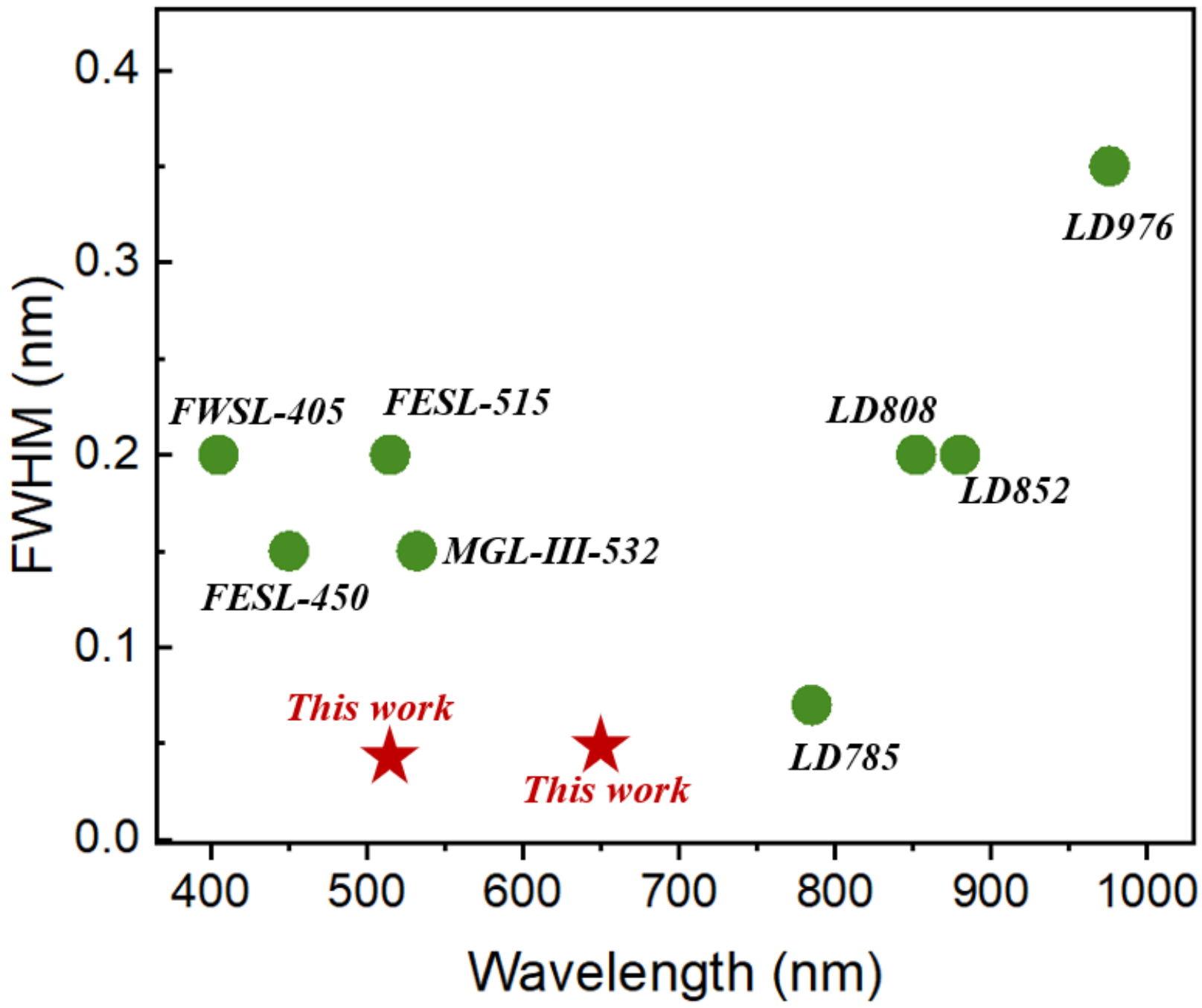

Fig. S1. Comparison of laser FWHM performance. Comparison of our laser's FWHM performance with that of commercial laser diodes.

Table S1. Performance Comparison: CNPs-liquid Laser vs. Commercial Laser Diodes

Company	Model	Cavity Type	Band	SMSR (dB)
Thorlabs	All models (over 100 types)	FP	VIS	＜30
	LD785	FP	NIR	25-40
	LD808			
	LD852			
	L976SEV1			
Frankfurt Laser Company	FWSL-405	FP	VIS	—
	FESL-450	DFB	VIS	~30
	FESL-515	DFB	VIS	~28
	FWSL-525	FP	VIS	—
Changchun New Industries Optoelectronics Technology Co., Ltd.	MGL-III-532	FP	VIS	~31
	MDL-III-405	FP	VIS	~30
**Our Work**	**CNPs-liquid laser**	**Vernier effect mode selection**	**VIS**	**31.1**

S7. Linear Tuning Analysis

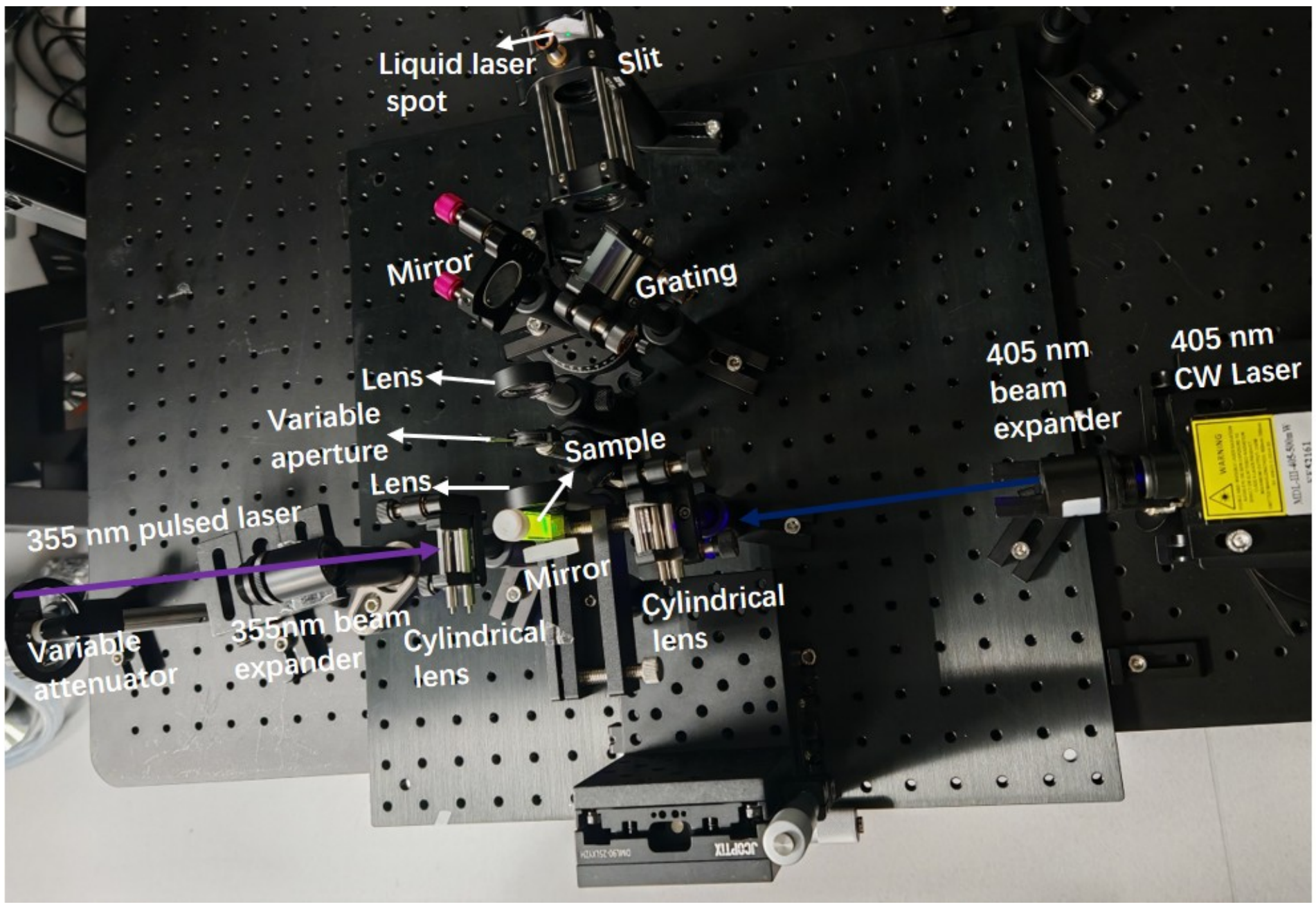

Fig. S2. CNP liquid laser setup. Photograph of the CNP liquid laser setup.

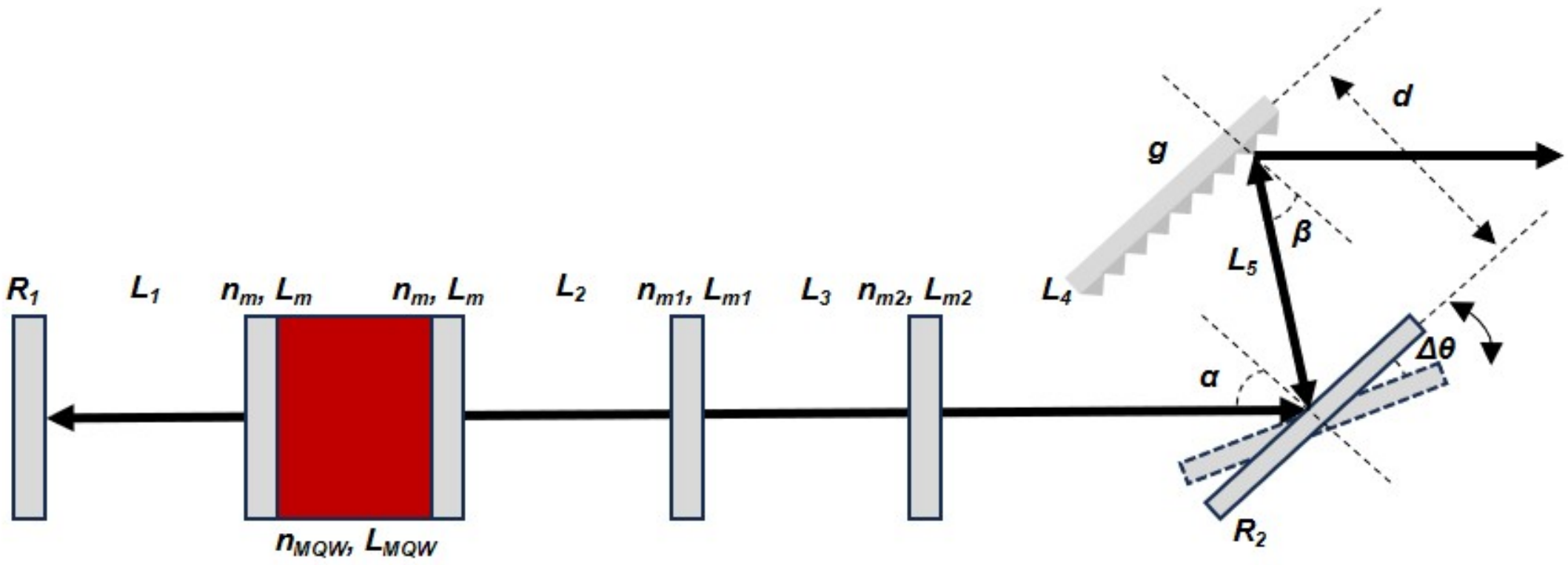

Fig. S3. Liquid external-cavity laser configuration. Schematic diagram of the liquid external-cavity laser.

Table S2. Parameter Summary

L_1	L_2	L_3	L_4	d	n_m	n_{m1},n_{m2}	n_{m1},n_{m2}	g
30 mm	60 mm	75 mm	100 mm	29 mm	1.46	1.515	1.49	416.67nm

A schematic diagram of the liquid external-cavity laser is shown in the Fig. S3. The laser cavity consists of the mirror on the left and the grating, with collimation achieved through lens L_{m1} and lens L_{m1} in the middle, and tuning performed via mirror

R_2. Ignoring the optical path changes during collimation, according to the phase-matching condition of the FP cavity,

$$\varphi = \frac{4\pi nL}{\lambda_0} = 2\pi m \tag{1}$$

The resonant wavelength of the laser cavity is defined by the following equation,

$$\begin{aligned} m\lambda_0 = 2nL_{total} &= 2(2(n_m L_m + n_{m1} L_{m1} + n_{m2} L_{m2}) \\ &+ n_{CNP} L_{CNP} + L_1 + L_2 + L_3 + L_4 + L_5) \end{aligned} \tag{2}$$

Where m is the laser mode number, λ_o represents the wavelength determined by the laser cavity; L_{total} is the total length of the laser cavity; n_{m} and L_{m} are the refractive index and the wall thickness of the cuvette, respectively; n_{CNP} and L_{CNP} represent the refractive index and the length of the CNPs, respectively; n_{mi} and L_{mi} (i=1,2) are the refractive index and the thickness of lens 1 and lens 2, respectively; L_{j} (j=1,2,3,4,5) represents the length of each segment of the laser cavity, where $L_5 = d/\cos(\beta)$, d is the spacing between mirror 2 and the grating, β is the first-order incident angle of the grating, which changes with the rotation angle $\Delta\theta$ of the mirror. The angle between the incident light and mirror 2 is α, with the initial incident angle being α_0, so $\alpha = \alpha_o + \Delta\theta$, and $\beta = \alpha_o + 2\Delta\theta$.

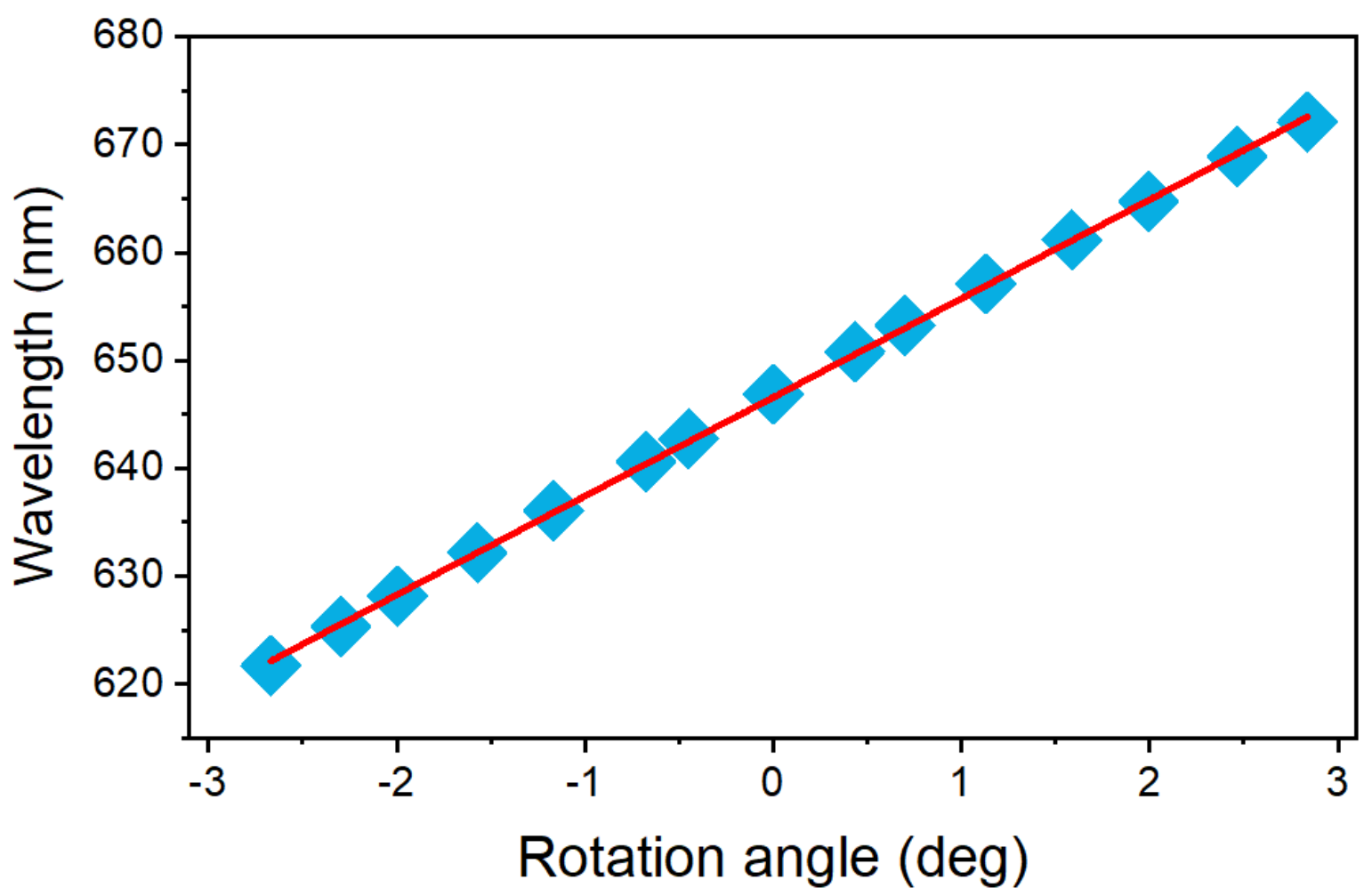

Fig. S4. Linear tuning of the laser output wavelength. Functional relationship between the mirror rotation angle and the output wavelength.

The resonant wavelength of the grating is defined by the following equation,

$$N\lambda_g = 2g\sin(\beta) \tag{3}$$

where $N = 1$ is the grating diffraction order, and g is the grating period. To achieve single-mode output during the tuning process, the condition $\lambda_0 = \lambda_g$ must be satisfied. In the experiment, the structural parameters of the laser cavity are listed in the table. The initial incident angle $\alpha_0 = 50.92°$, and the initial laser wavelength is 646.9 nm, as shown in the figure. By varying $\Delta\theta$ within the range of [-2.67°, 2.83°], a wide tuning range of 50.3 nm was achieved, along with good linear tuning performance. The tuning efficiency was 9.15 nm/deg, and the tuning range was limited by the gain bandwidth.

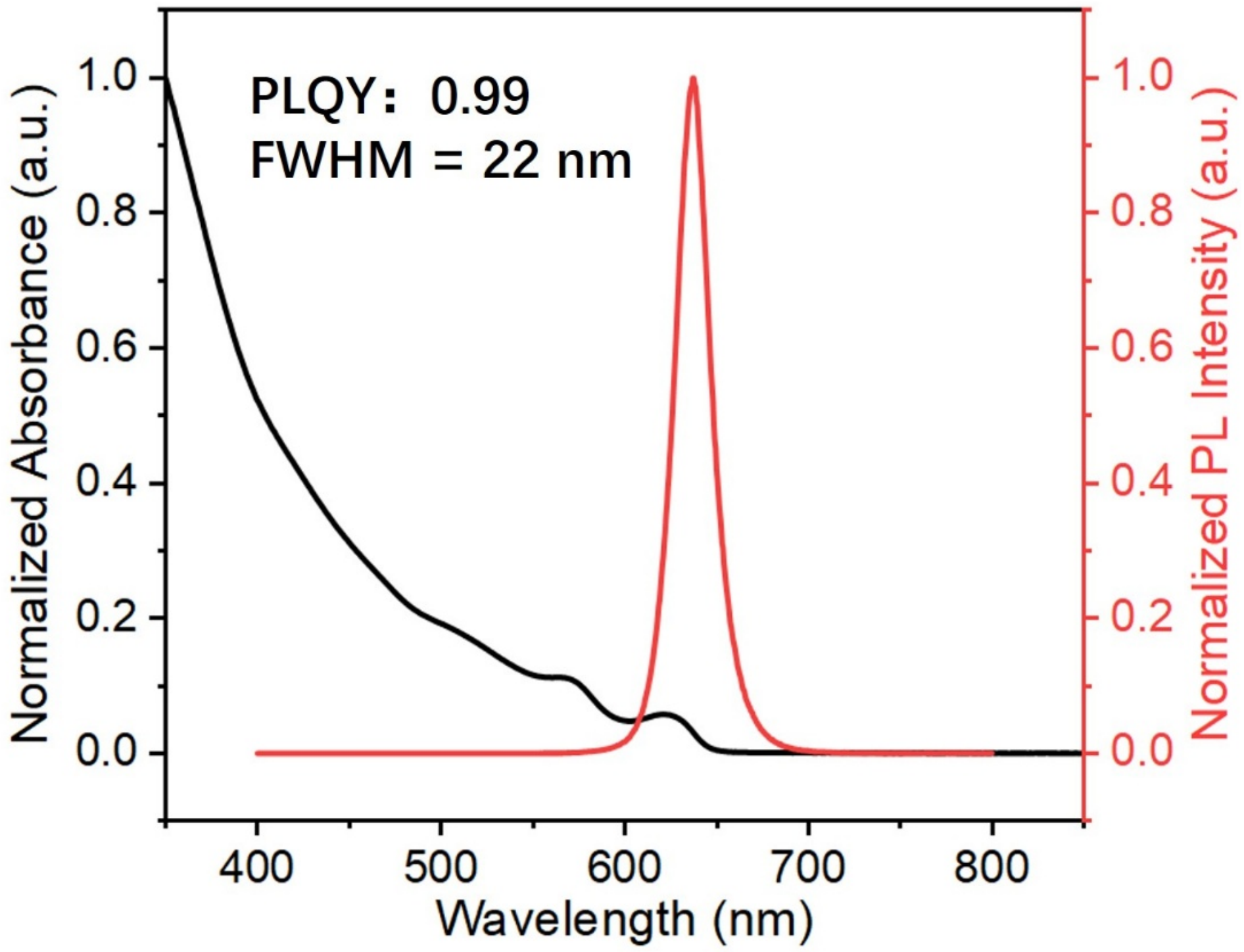

Fig. S5. Optical spectra and PLQY of CdSe/CdSeS/CdZnS CNPs. Normalized absorbance and photoluminescence spectra of the as CdSe/CdSeS/CdZnS core/crown/shell CNPs and its PLQY.

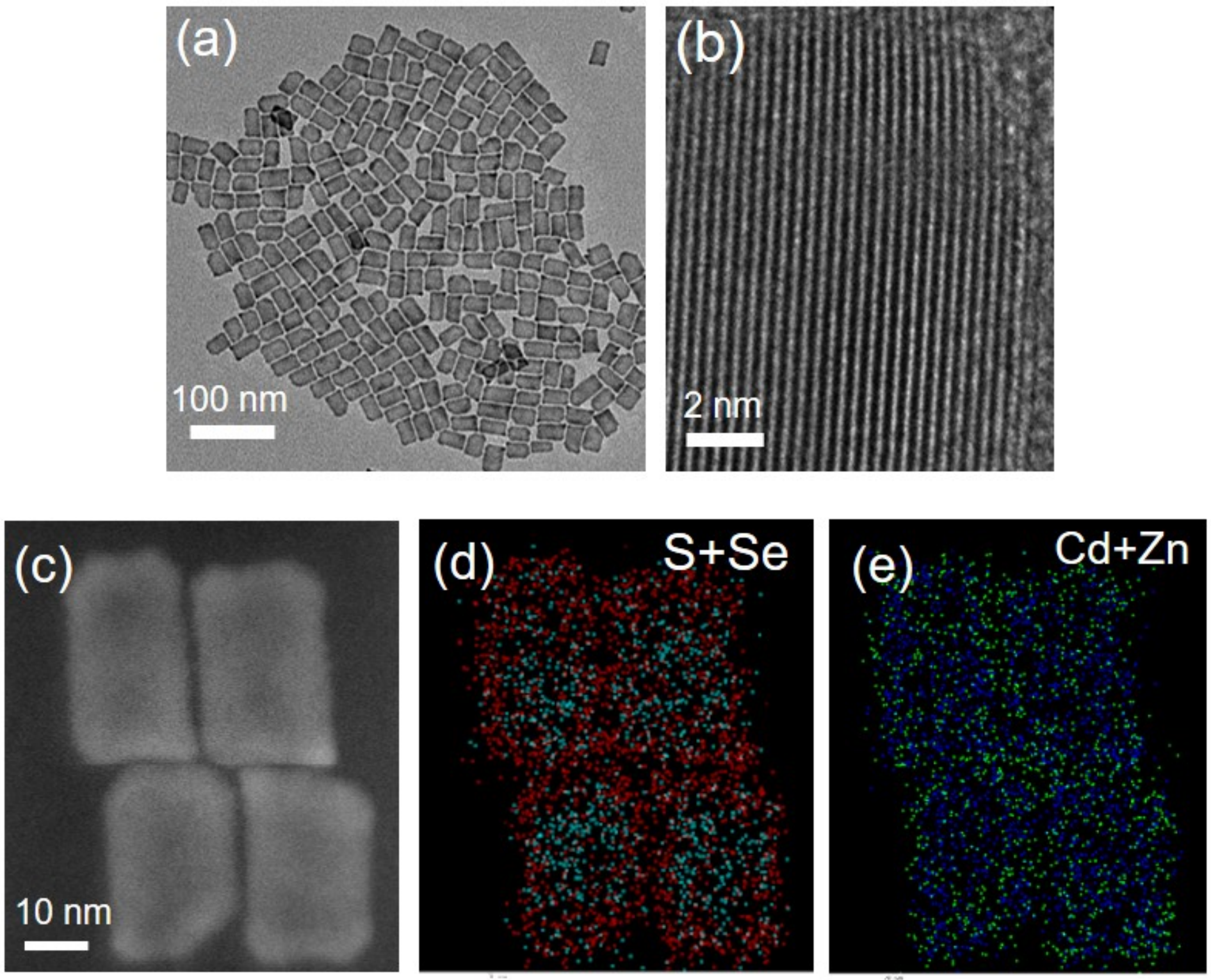

Fig. S6. Structural and elemental characterization of CdSe/CdSeS/CdZnS CNPs. (a) Bright-field TEM image of CdSe/CdSeS/CdZnS core/crown/shell CNPs. (b) High-magnification TEM image of a single CNP. (c) TEM image of four CNPs. (d) EDS elemental mapping of S (red) and Se (cyan). (e) EDS elemental mapping of Cd (blue) and Zn (green). For the S and Zn elements, they are mainly distributed on the outer side of the CNPs, which aligns with the designed core/crown/shell structure.

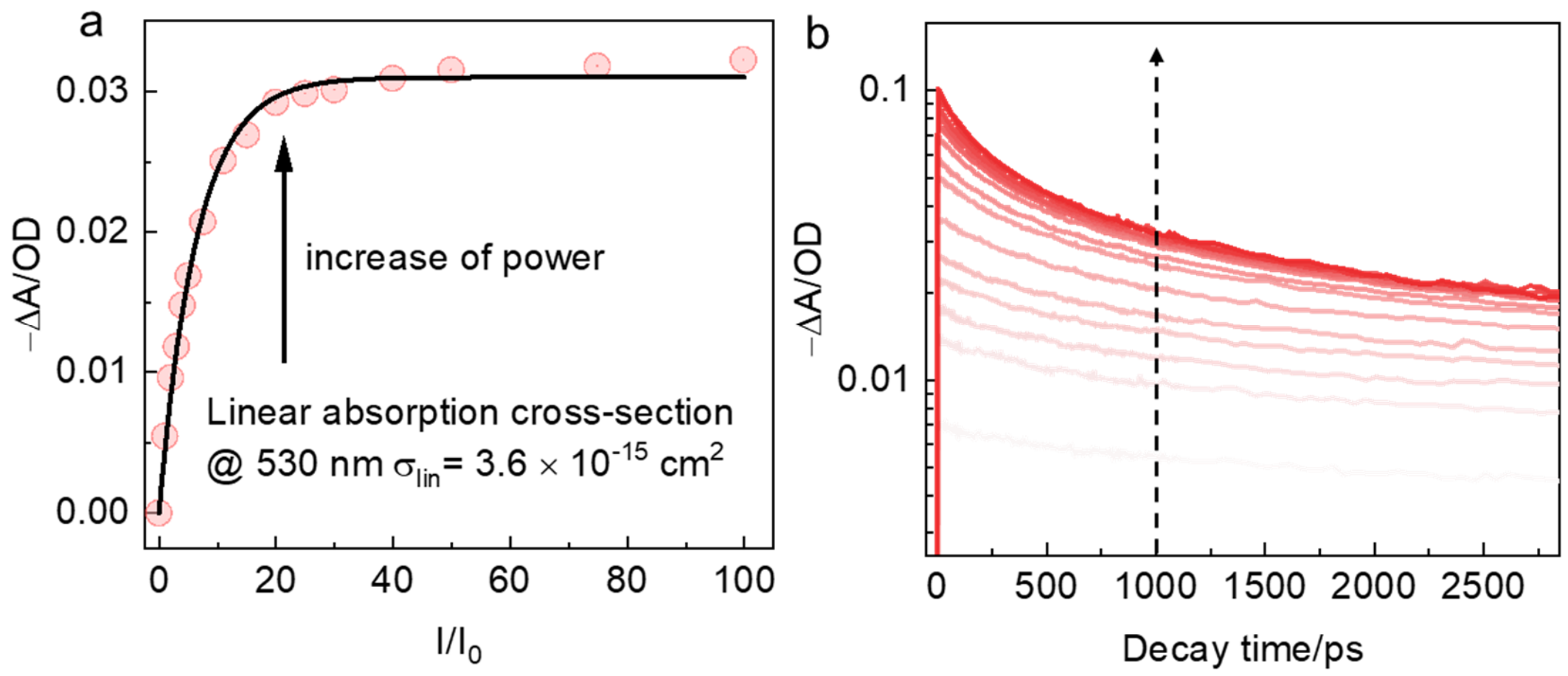

Fig. S7. Excitation-intensity-dependent GSB dynamics. (a) GSB signal amplitude at a time delay of 1 ns as a function of excitation intensity of 636 nm. (b) Excitation intensity-dependent decay curves of 636 nm.

The solid curves are the best fitting lines based on equation*(34)*:

$$-A\left(\frac{\mathrm{I}}{\mathrm{I}_0}\right) = -A_{\max}[1 - e^{-(\mathrm{I}/\mathrm{I}_0)\cdot\sigma_{\mathrm{lin}}\cdot I_0}] \tag{4}$$

where $A\left(\frac{\mathrm{I}}{\mathrm{I}_0}\right)$ denotes the GSB signal amplitude of samples at 1 ns time delay as a function of excitation intensity, σ_{lin} is the linear absorption cross-sections and I_0 is the minimum excitation intensity used in the fs-TA experiment.

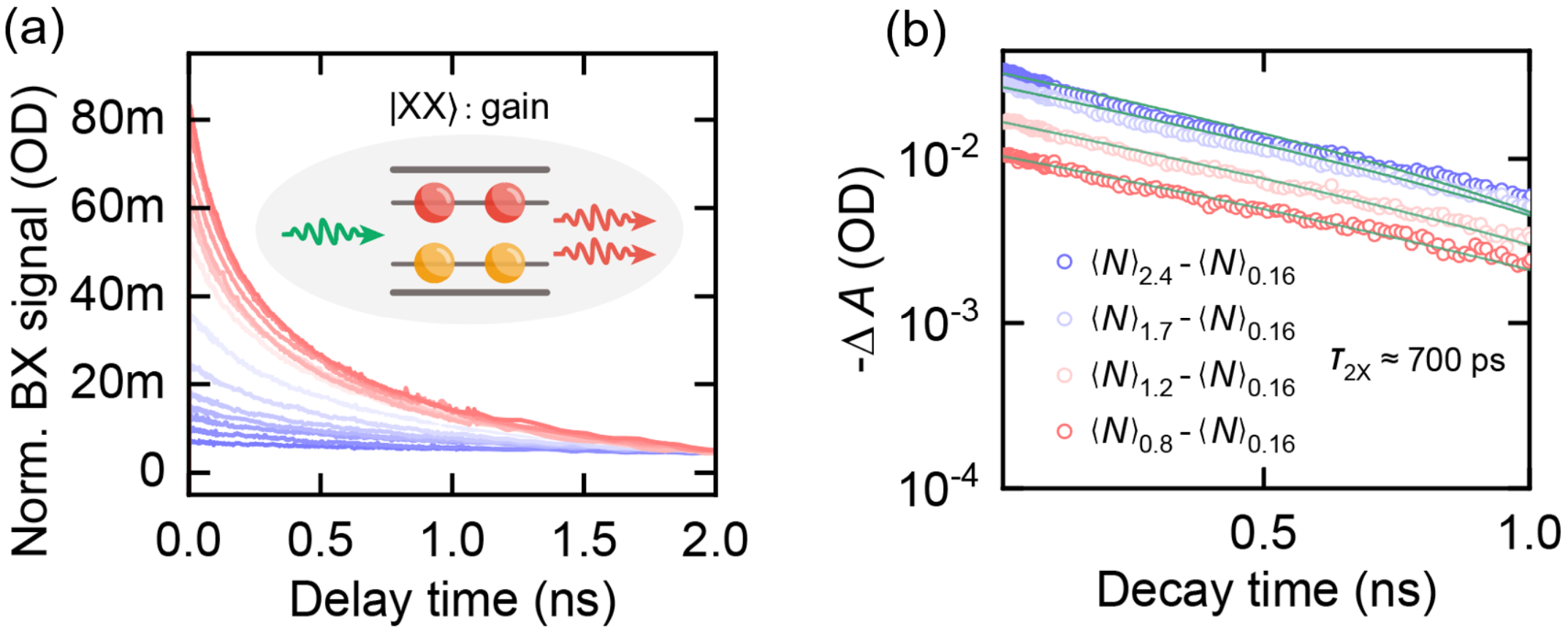

Fig. S8. Transient-absorption kinetics and biexciton recombination. (a) TA kinetic traces probed at 636 nm for varying $\langle N_0 \rangle$. (b) The biexciton recombination kinetics obtained by subtracting the kinetic trace of $\langle N_0 \rangle \approx 0.16$ from that of $\langle N_0 \rangle \approx 0.8$, 1.2, 1.7 and 2.4, and their fit to single-exponential decay with $\tau_{XX} = 700$ ps.

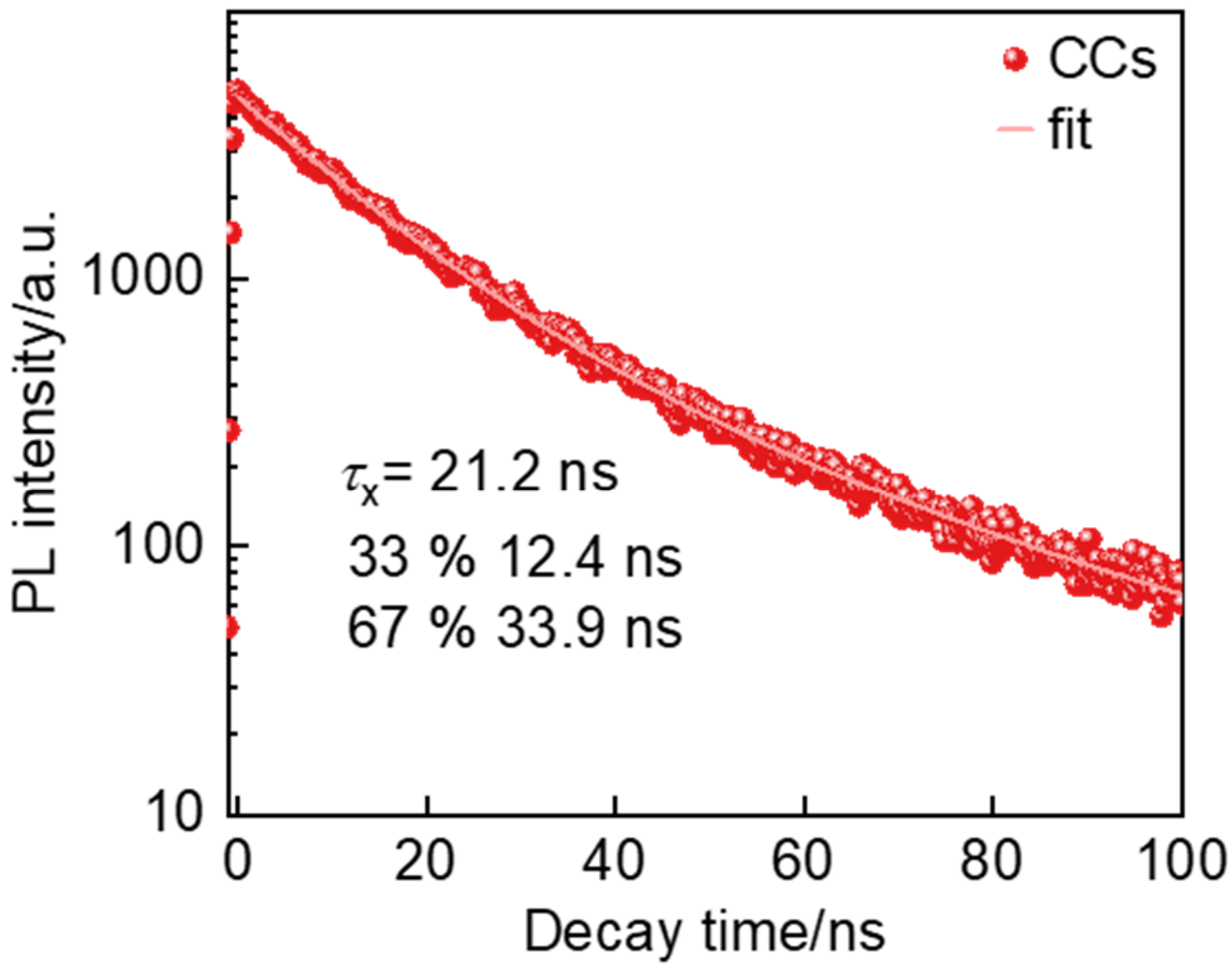

Fig. S9. Time-resolved photoluminescence dynamics of CdSe/CdSeS/CdZnS CNPs. The time-resolved PL kinetic curve of CdSe/CdSeS/CdZnS core/crown/shell CNPs dispersed in hexane and its biexponential fitting. The lifetime components and the average single-exciton lifetime (τ_X) are specified.

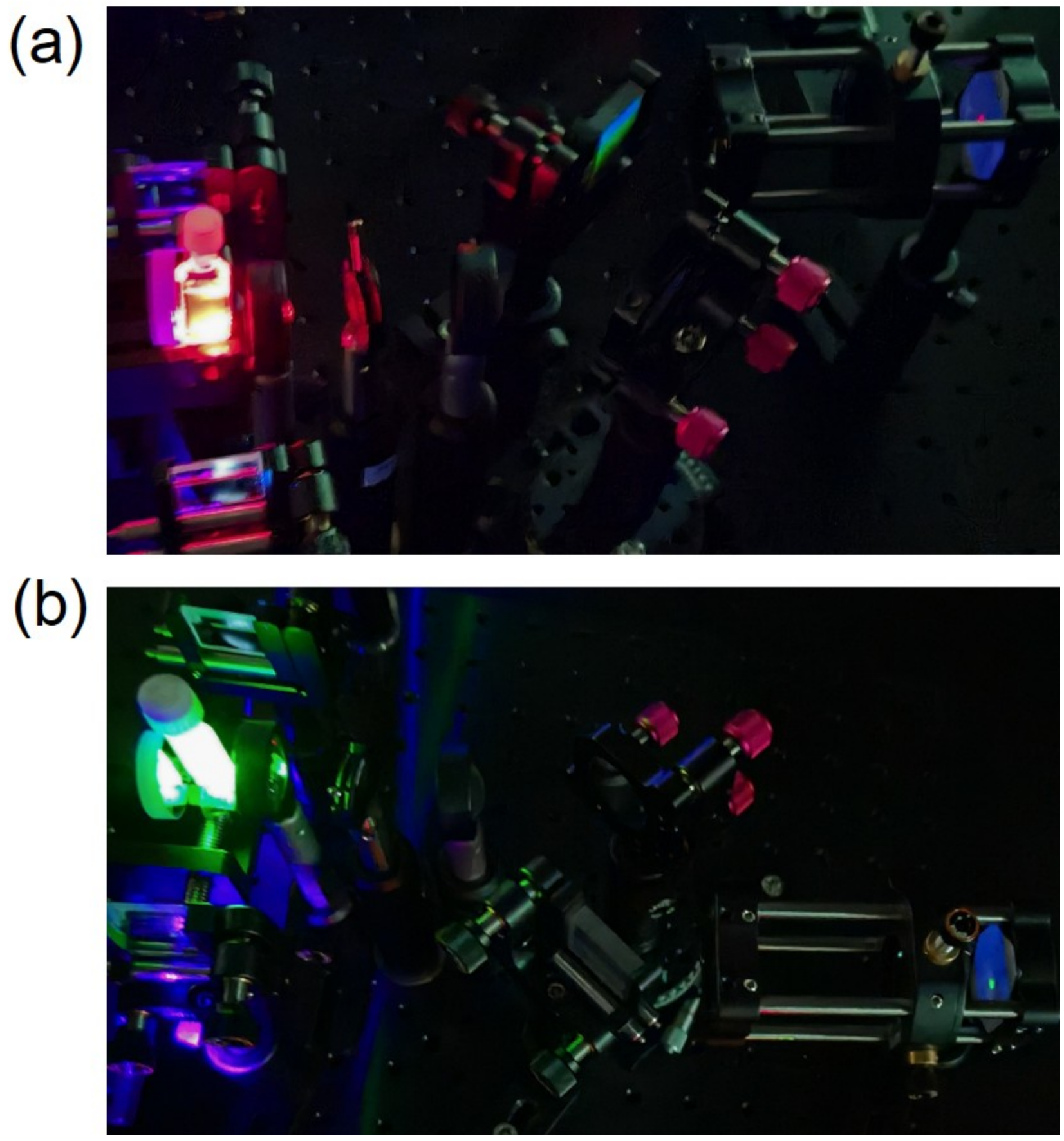

Fig. S10. Photographs of two CNP liquid-laser systems. Photographs of the CdSe/CdSeS/CdZnS core/graded crown/graded shell CNP liquid laser and the CdSe/CdSeS core/alloyed-crown CNP liquid laser.

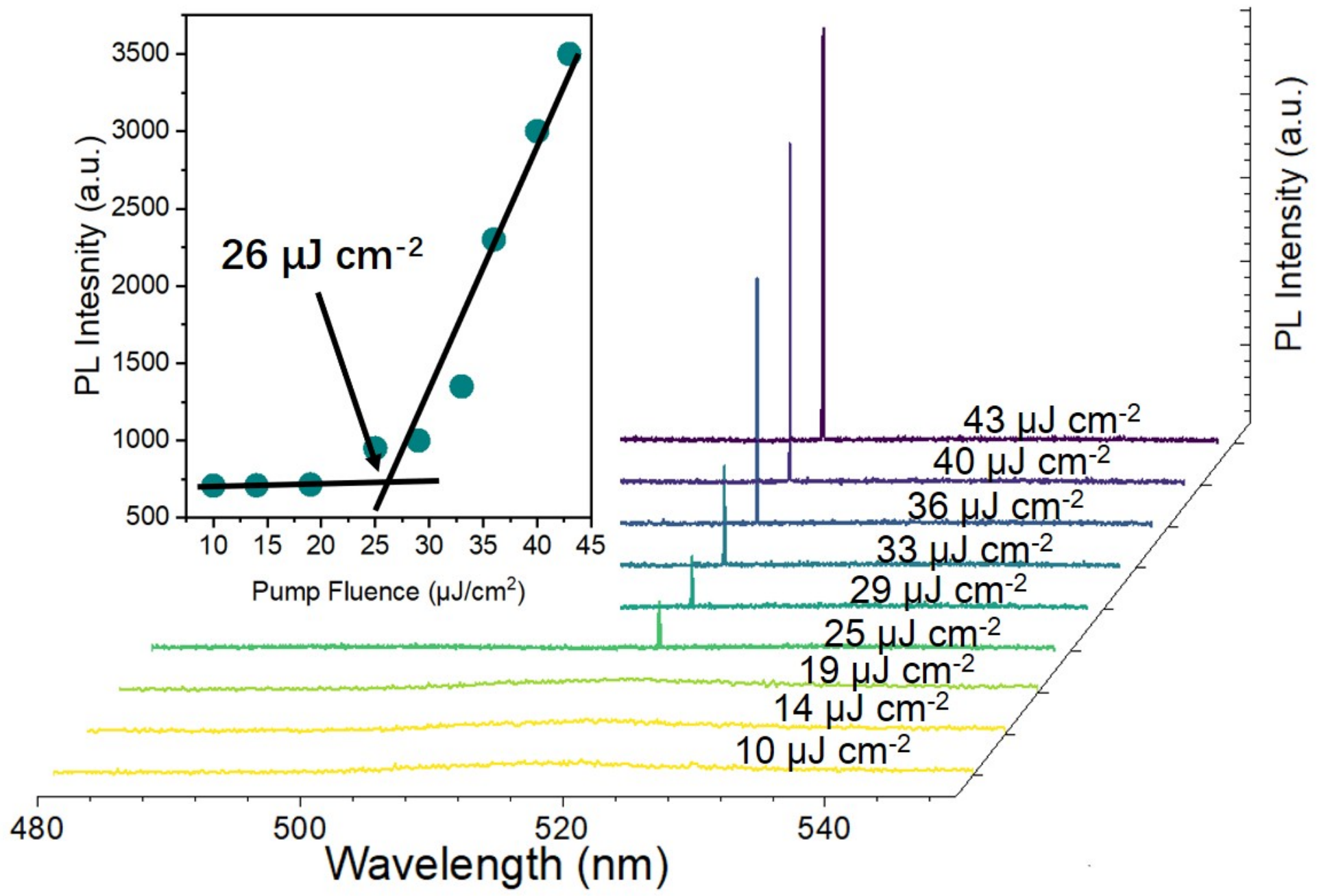

Fig. S11. Pump-fluence-dependent emission of the CNP liquid laser. Evolution of the emission spectrum of the CdSe/CdSeS core/alloyed-crown CNP liquid laser as a function of pump fluence. The inset shows the laser threshold fitted to be 26 μJ cm^{-2}.

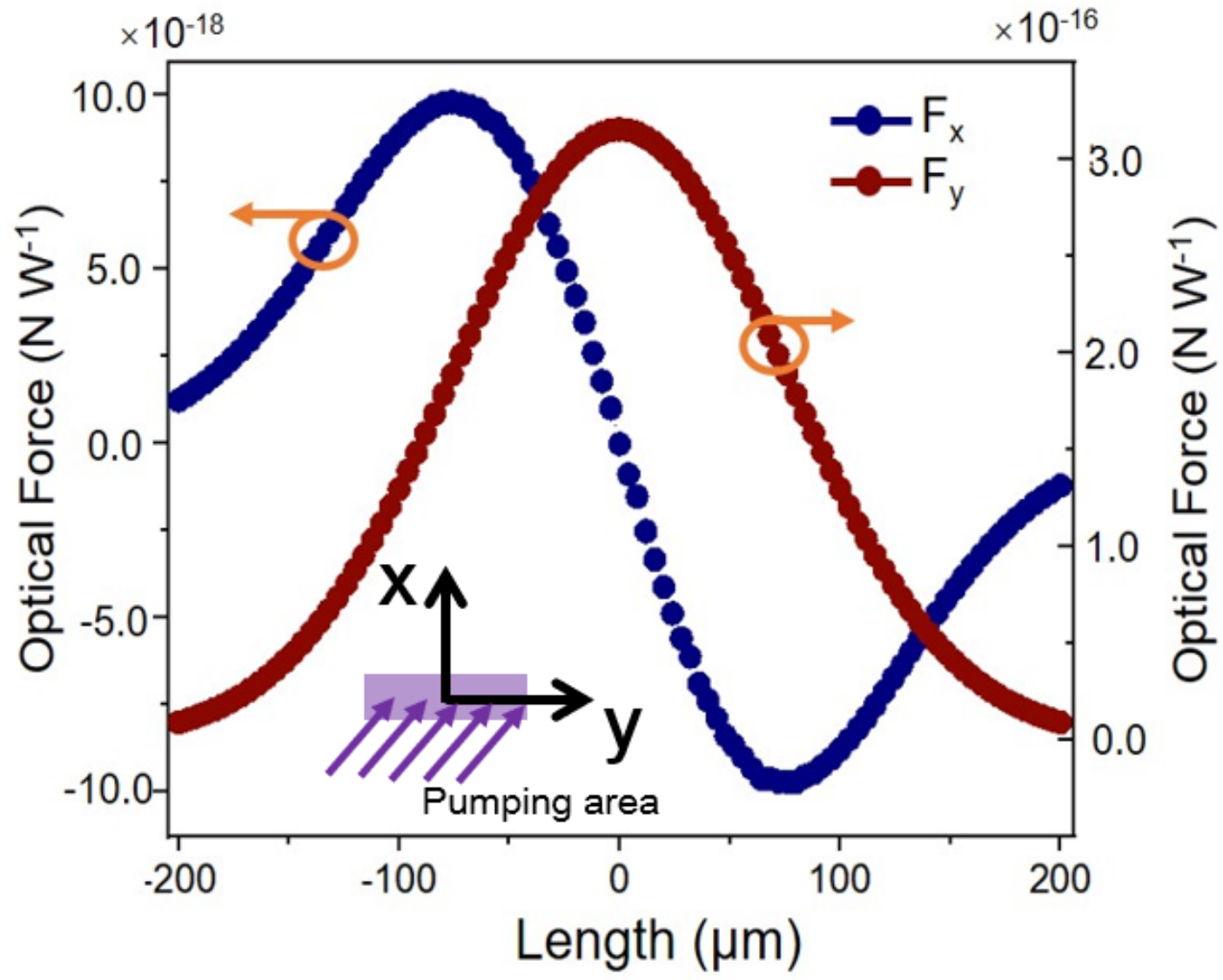

Fig. S12. Optical gradient forces along the x and y directions. Optical gradient forces experienced by the CNPs along the x and y directions within the pump region.

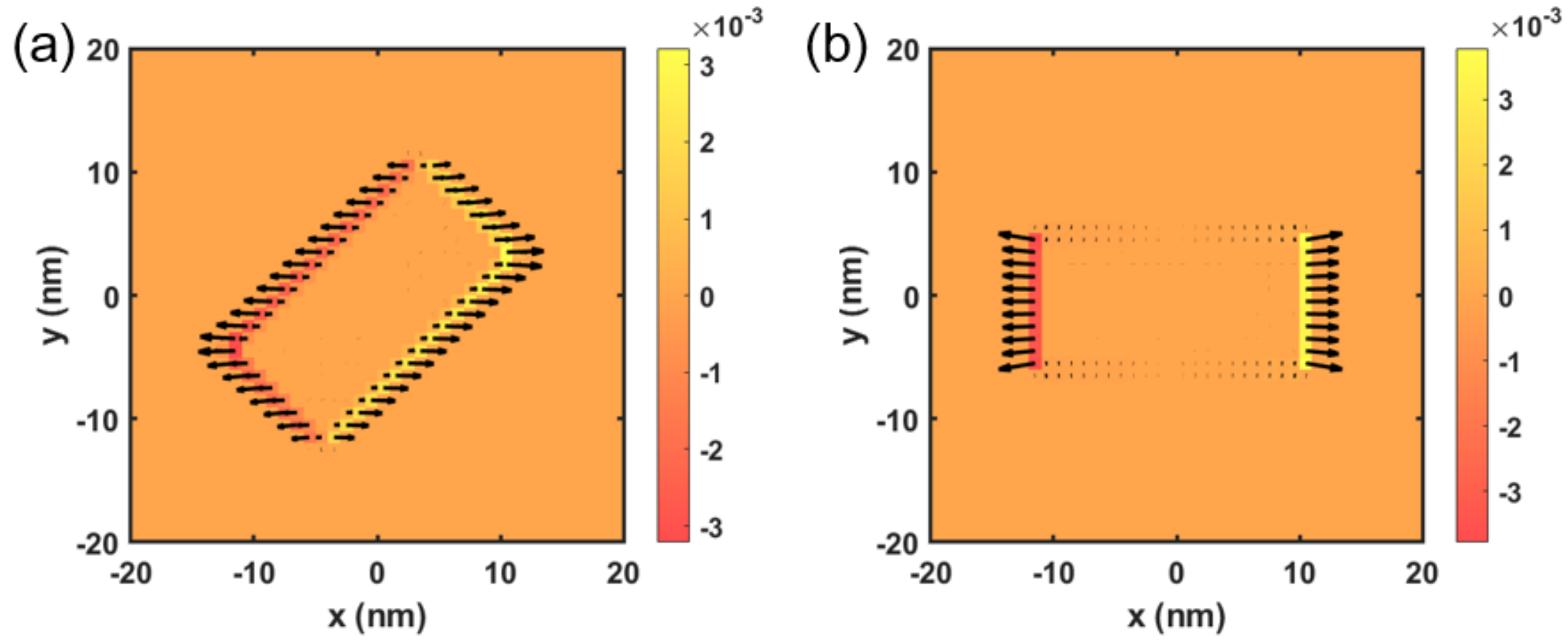

Fig. S13. Orientation-dependent optical gradient forces on CNPs. (a) Optical gradient forces acting on the CNPs at a rotation angle of oz = 45°. (b) Optical gradient forces acting on the CNPs at a rotation angle of oz = 0°. The color of the scale bar and the length of the force arrows represent the direction and magnitude of the applied forces.

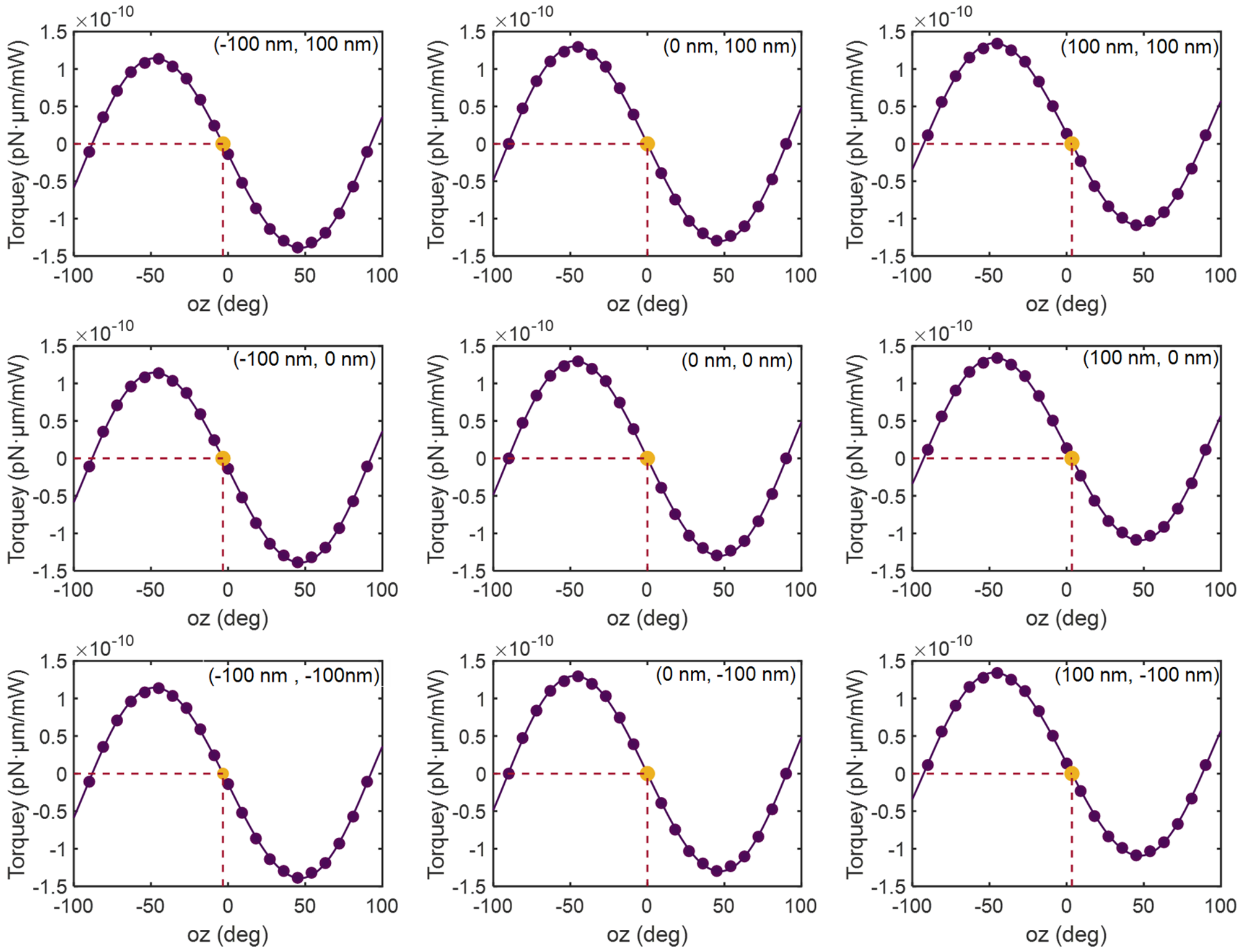

Fig. S14. Position-dependent orientational equilibrium of CNPs in the optical field. Calculated optical torque as a function of the orientation angle oz at nine representative spatial positions around the beam center, as indicated in each panel. The purple curves and symbols represent the calculated torque, while the orange circles mark the zero-torque equilibrium orientations, with the red dashed lines serving as guides to the eye. The negative slope of the torque curves at the zero crossings indicates stable orientational equilibria. Although the equilibrium angle is approximately 0° at the beam center, slight position-dependent shifts of several degrees occur away from the center, indicating that the CNPs remain close to their preferred orientation while retaining small angular deviations in the final quasi-equilibrium state.

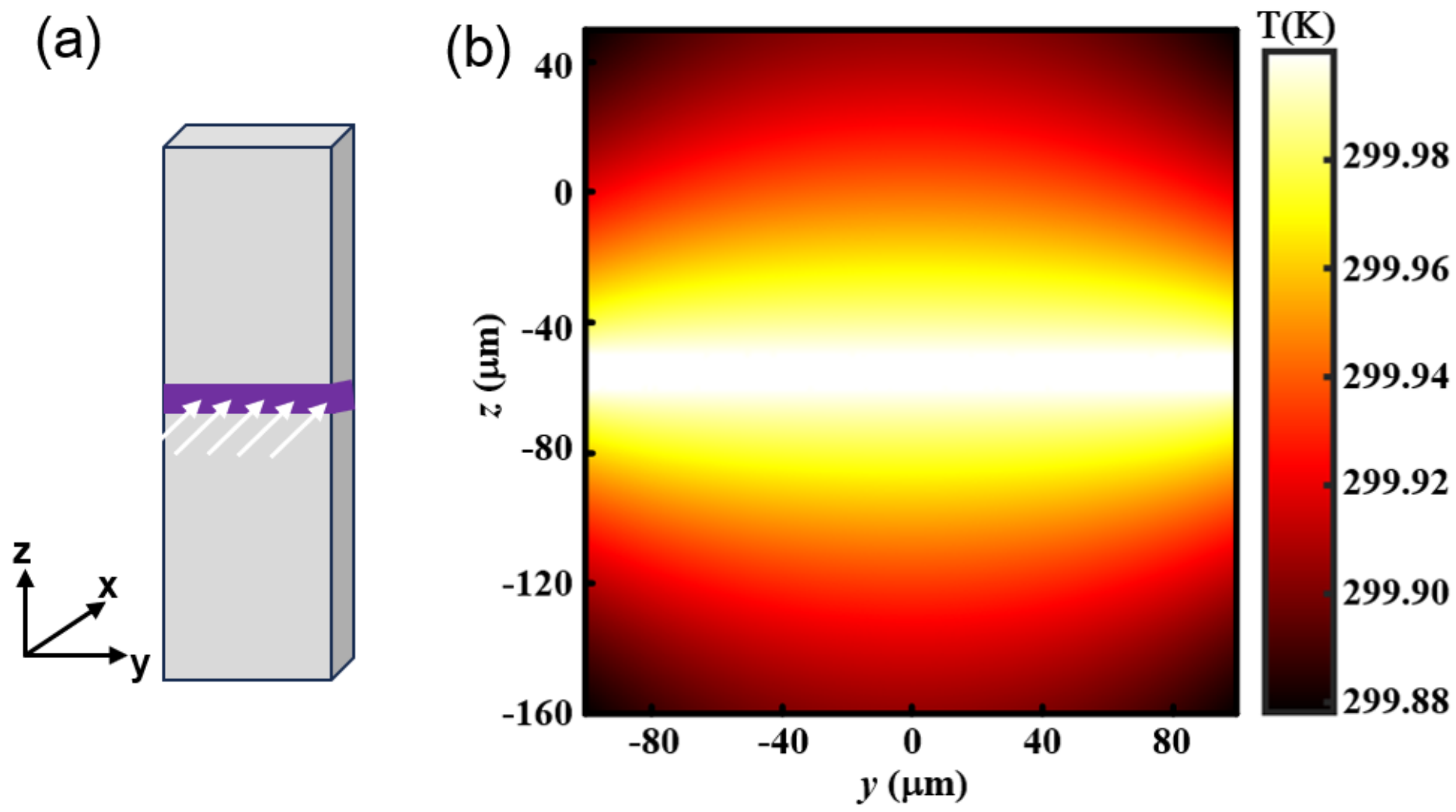

Fig. S15. Temperature distribution in the CNP fluid system. (a) Illustration of the xyz coordinate axes indicating the spatial orientation of the CNPs fluid system. (b) Temperature distribution of the CNPs fluid system after illumination in the y–z plane. The temperature gradually diffuses toward both sides, forming a non-uniform thermal gradient that induces laminar flow.

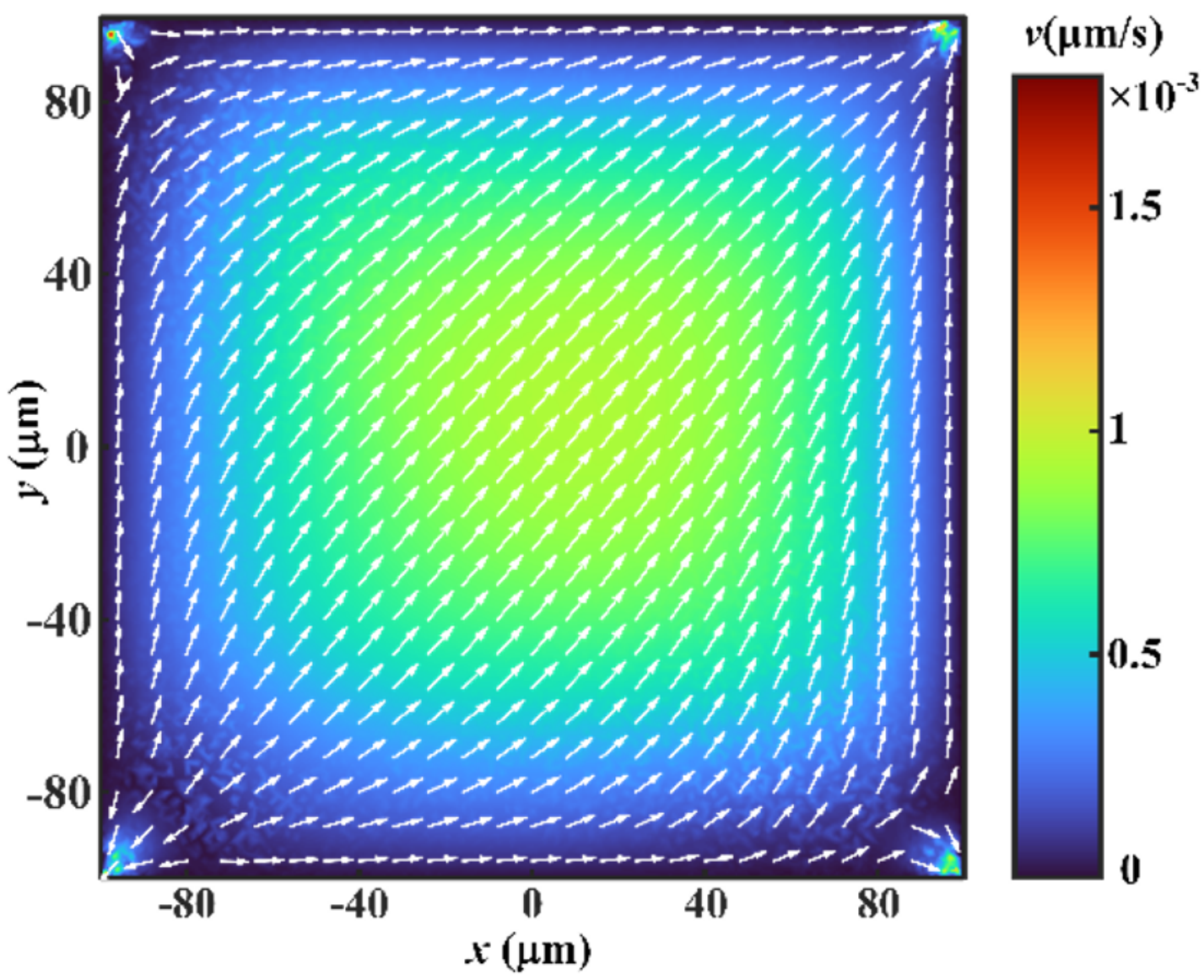

Fig. S16. Laminar-flow distribution in the CNP fluid system. Laminar flow distribution of the CNPs fluid system in the x–y plane, where the arrow directions indicate the flow orientation. The xyz coordinate axes indicating the spatial orientation are shown in Fig. S15.

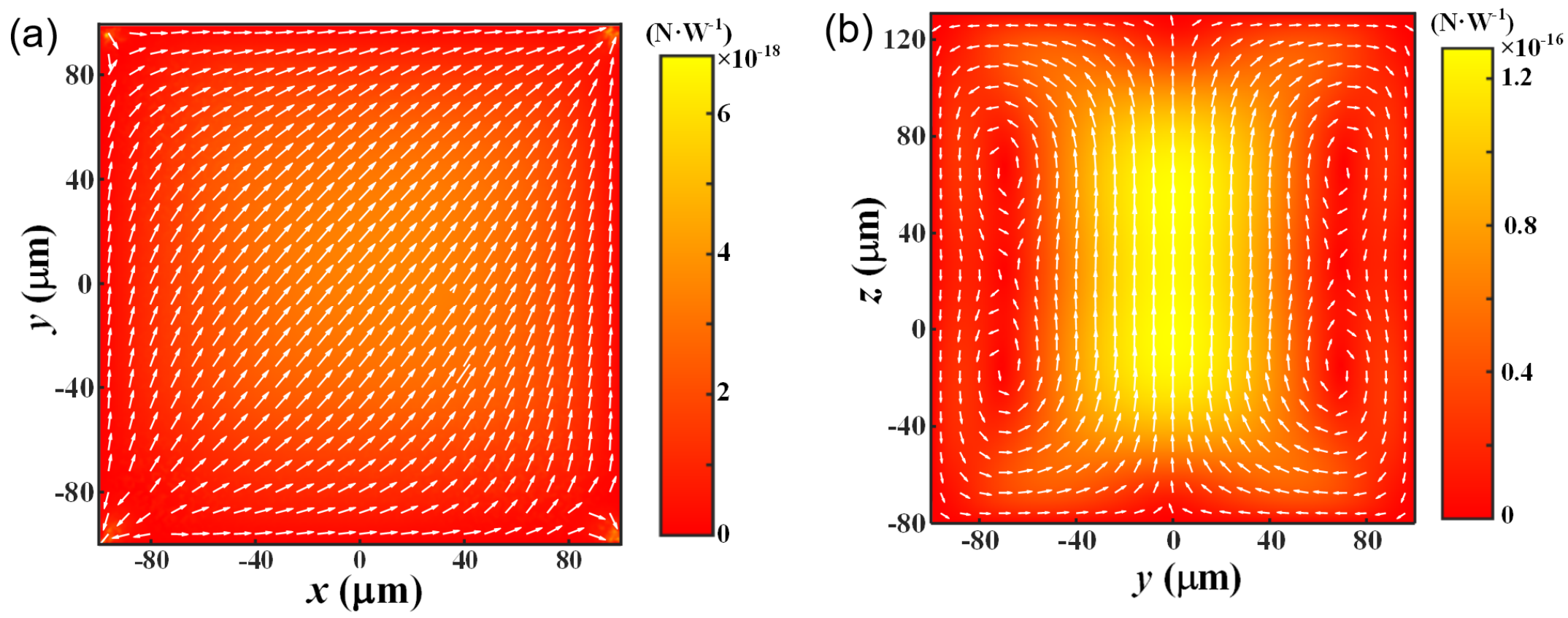

Fig. S17. Stokes-drag-force analysis of CNPs induced by thermally driven convection. (a) Stokes-drag-force distribution acting on CNPs in the x–y plane. (b) Stokes-drag-force distribution acting on CNPs in the y–z plane. The x-y-z coordinate axes defining the spatial orientation are shown in Fig. S15.

To quantify the thermally induced fluidic contribution, we estimated the hydrodynamic force acting on the CNPs, which is dominated by Stokes drag *(57)*:

$$\boldsymbol{F} = 6\pi\mu \boldsymbol{f}_{shape} \boldsymbol{R}\left(\overrightarrow{\boldsymbol{v}_1} - \overrightarrow{\boldsymbol{v}_2}\right)$$

where μ is the dynamic viscosity of the solution, $\boldsymbol{R}$ is the effective radius of the CNPs, $\vec{\boldsymbol{v}}_1$ 和 $\vec{\boldsymbol{v}}_2$ are the translational velocities of the solution and CNPs, respectively, and $\boldsymbol{f}$ is a geometry-dependent shape factor. For CNPs with dimensions of 30 × 20 × 5 nm^3, we used an average shape factor of $\boldsymbol{f}$ = 1.19, obtained from the directional values of 1.08, 1.12, and 1.37.

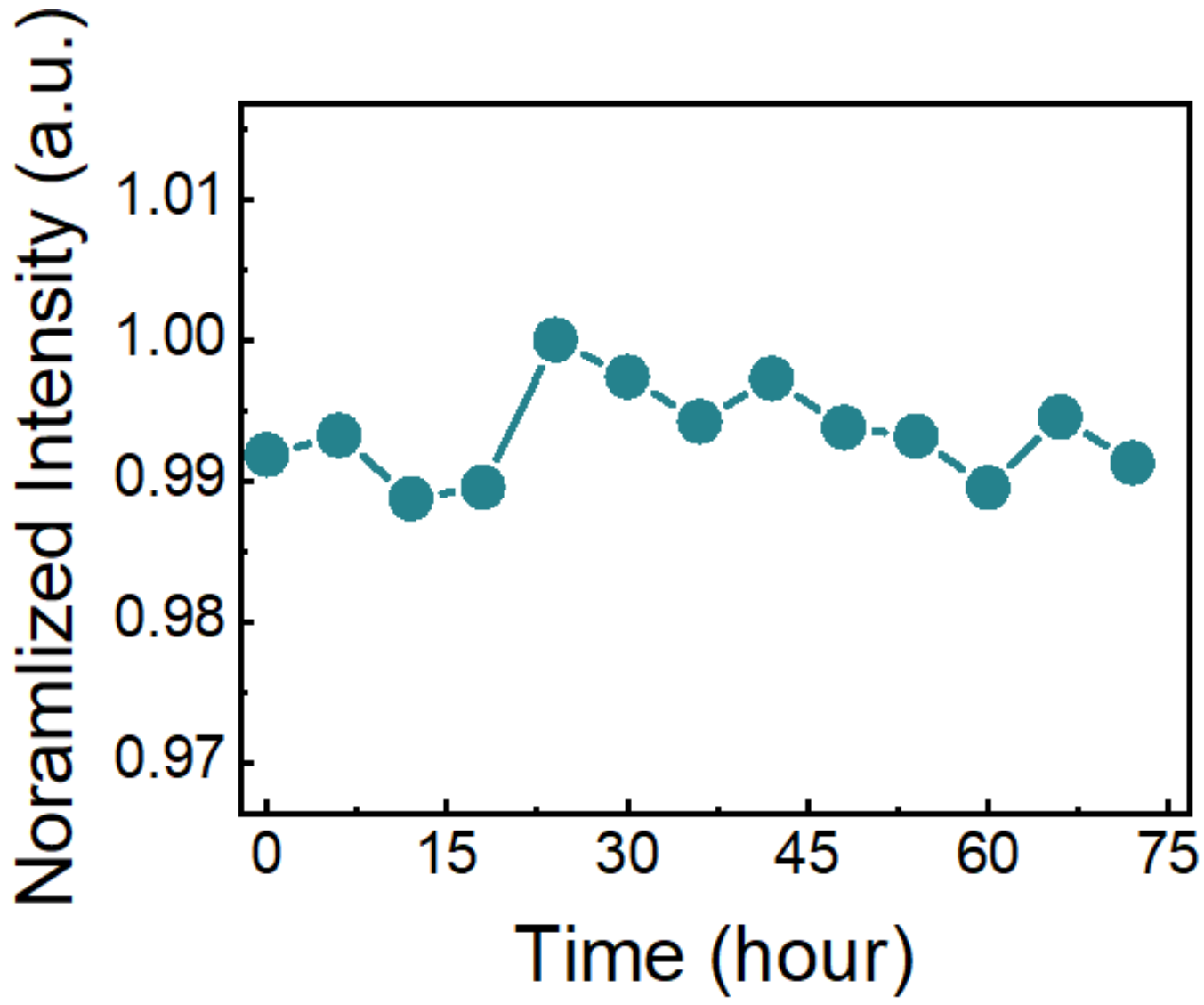

Fig. S18. 72 h output stability test of CNPs.

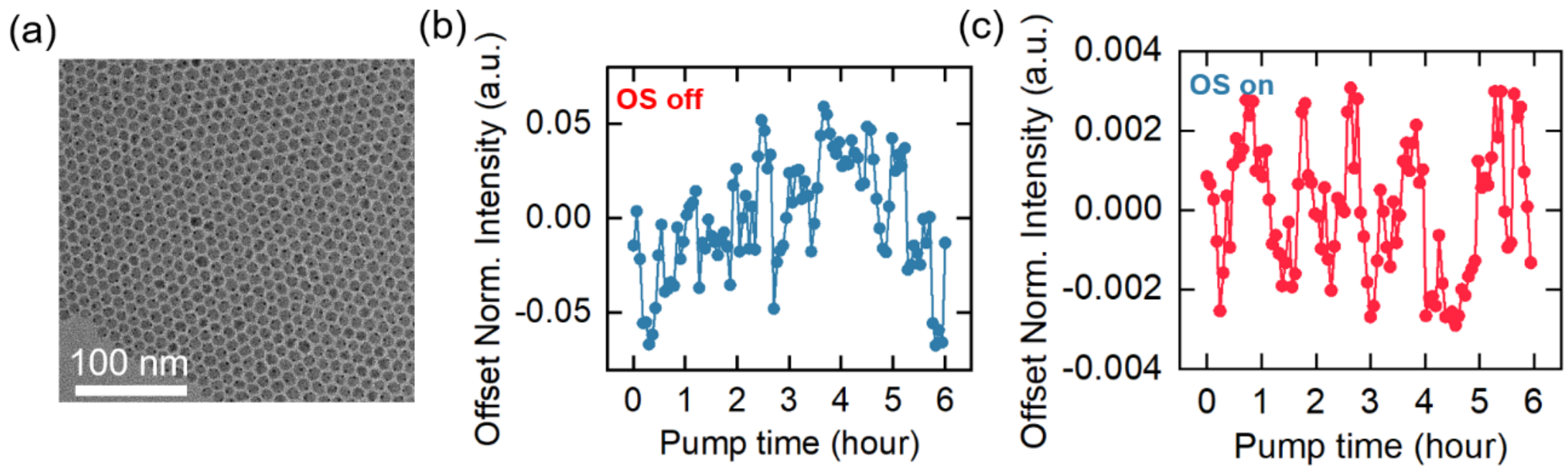

Fig. S19. Optofluidic stabilization of spherical perovskite CQDs. (a) TEM image of spherical perovskite CQDs. (b) Laser output intensity versus pump time for the CQD gain medium without optical tweezers. (c) Laser output intensity versus pump time for the CQD gain medium with optical tweezers enabled, demonstrating optofluidic self-stabilization.

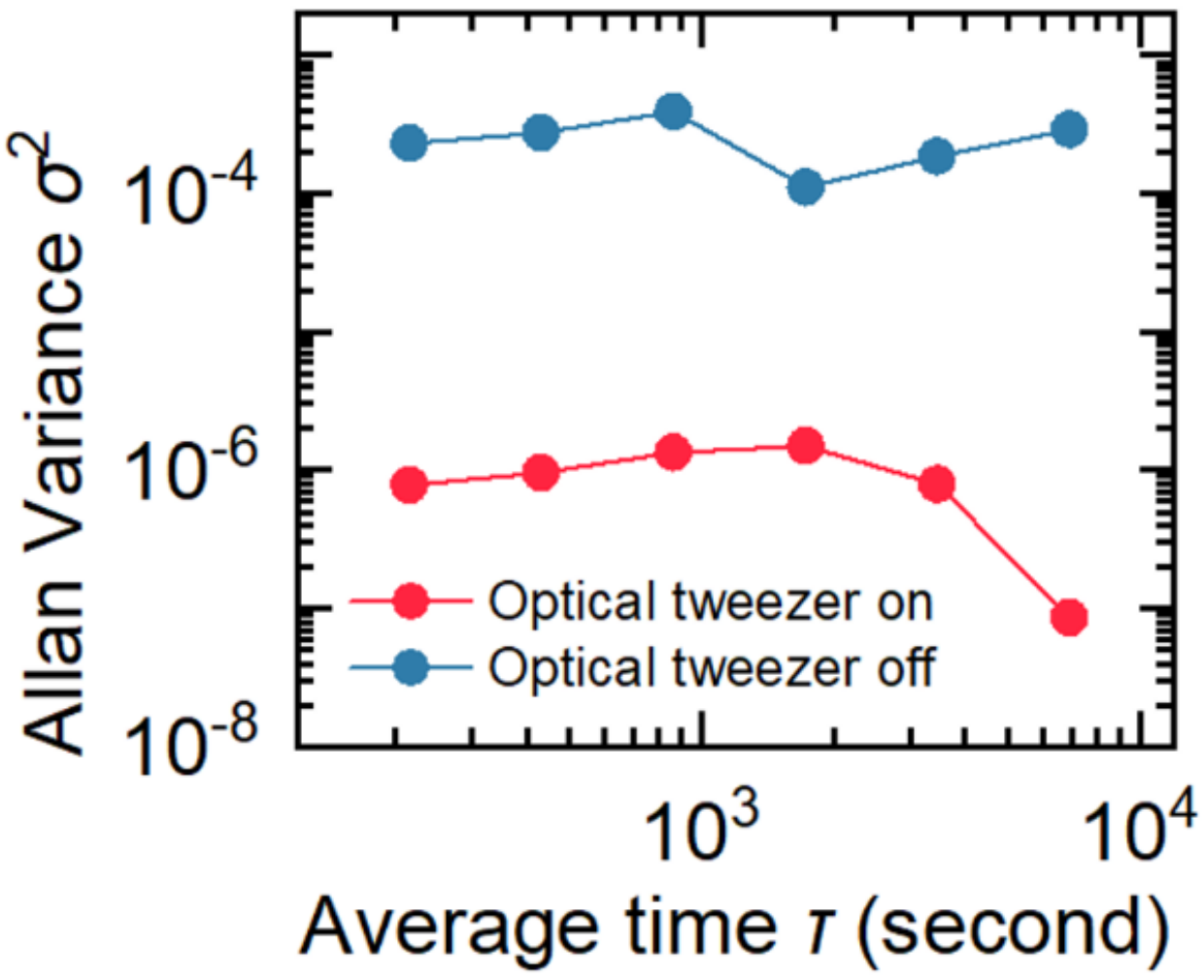

Fig. S20. Allan variance of the CQD laser output. Allan variance of the CQD laser output intensity with and without optical tweezers.

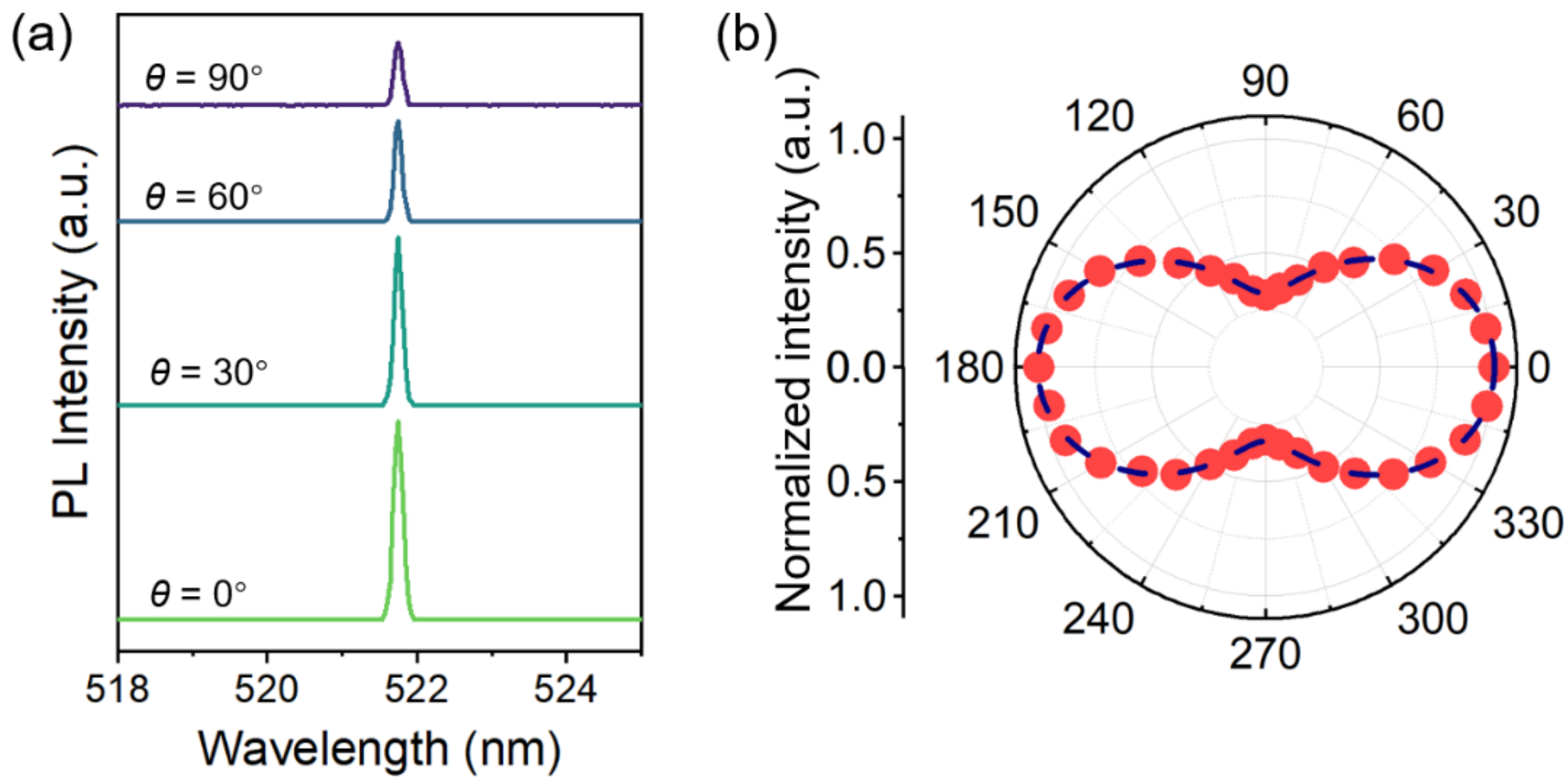

Fig. S21. Polarization characteristics of the CQD liquid laser. (a) Output spectra of the CQD laser at different polarizer rotation angles. (b) Integrated lasing-peak intensity as a function of the polarizer rotation angle.

Using near-spherical perovskite CQDs as an approximately isotropic reference system, we further evaluated whether the auxiliary CW beam can induce optofluidic stabilization in the absence of pronounced structural anisotropy. Under otherwise

identical optical and convective conditions, the CQD laser exhibited improved intensity stability after the auxiliary CW beam was enabled, confirming that optical-force-induced redistribution and thermally driven convection provide a general stabilization channel. However, the degree of polarization of the CQD laser was only 0.520, much lower than the value of 0.975 obtained for the CNP laser. This comparison indicates that CNP anisotropy mainly introduces an additional orientational-locking pathway, which is responsible for the highly polarized laser output.

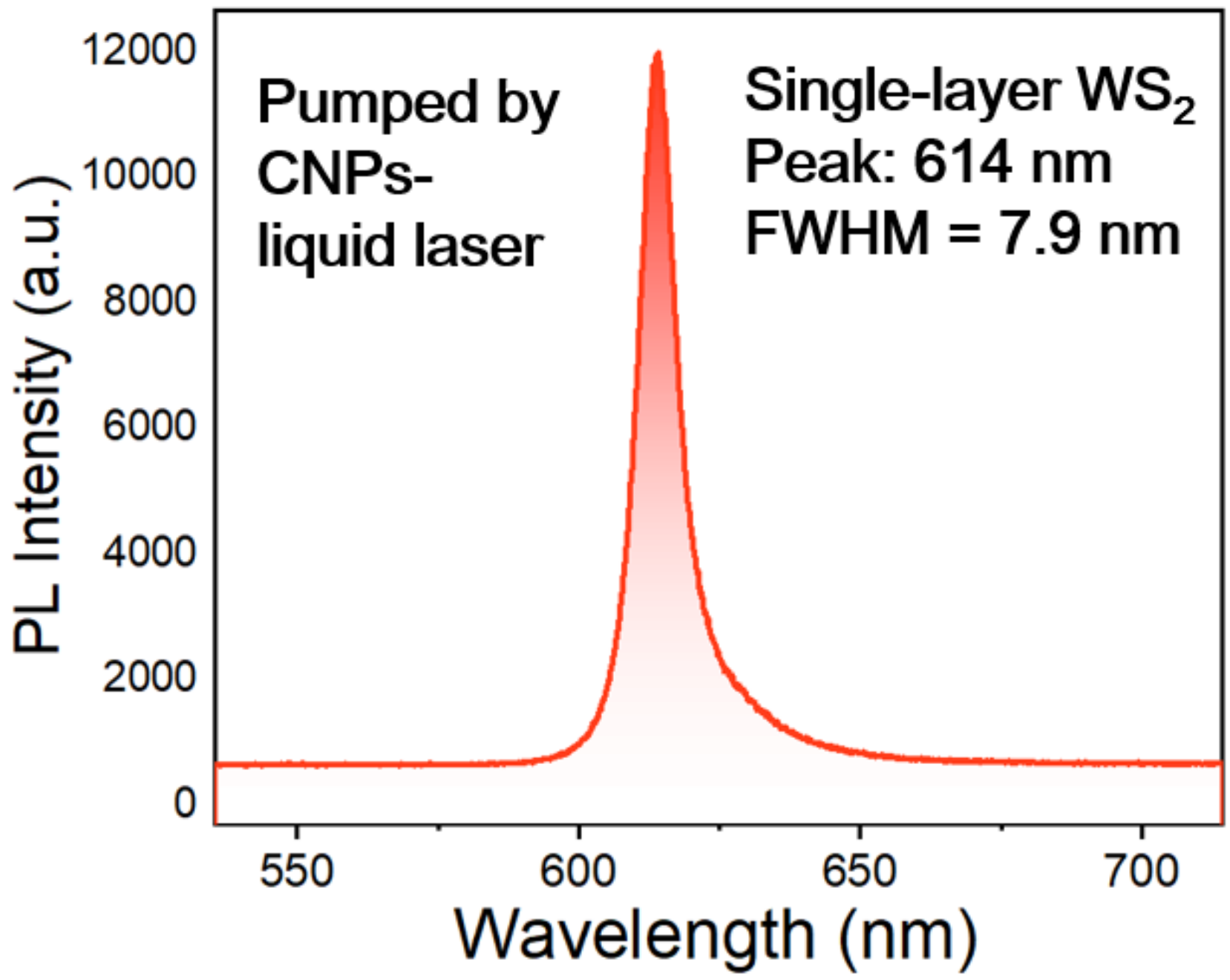

Fig. S22. Emission of monolayer WS_2 pumped by a CNP liquid laser. Typical emission spectrum of monolayer WS_2 pumped by a CNPs liquid laser, showing a peak at 614 nm with a full width at half maximum (FWHM) of 7.9 nm.

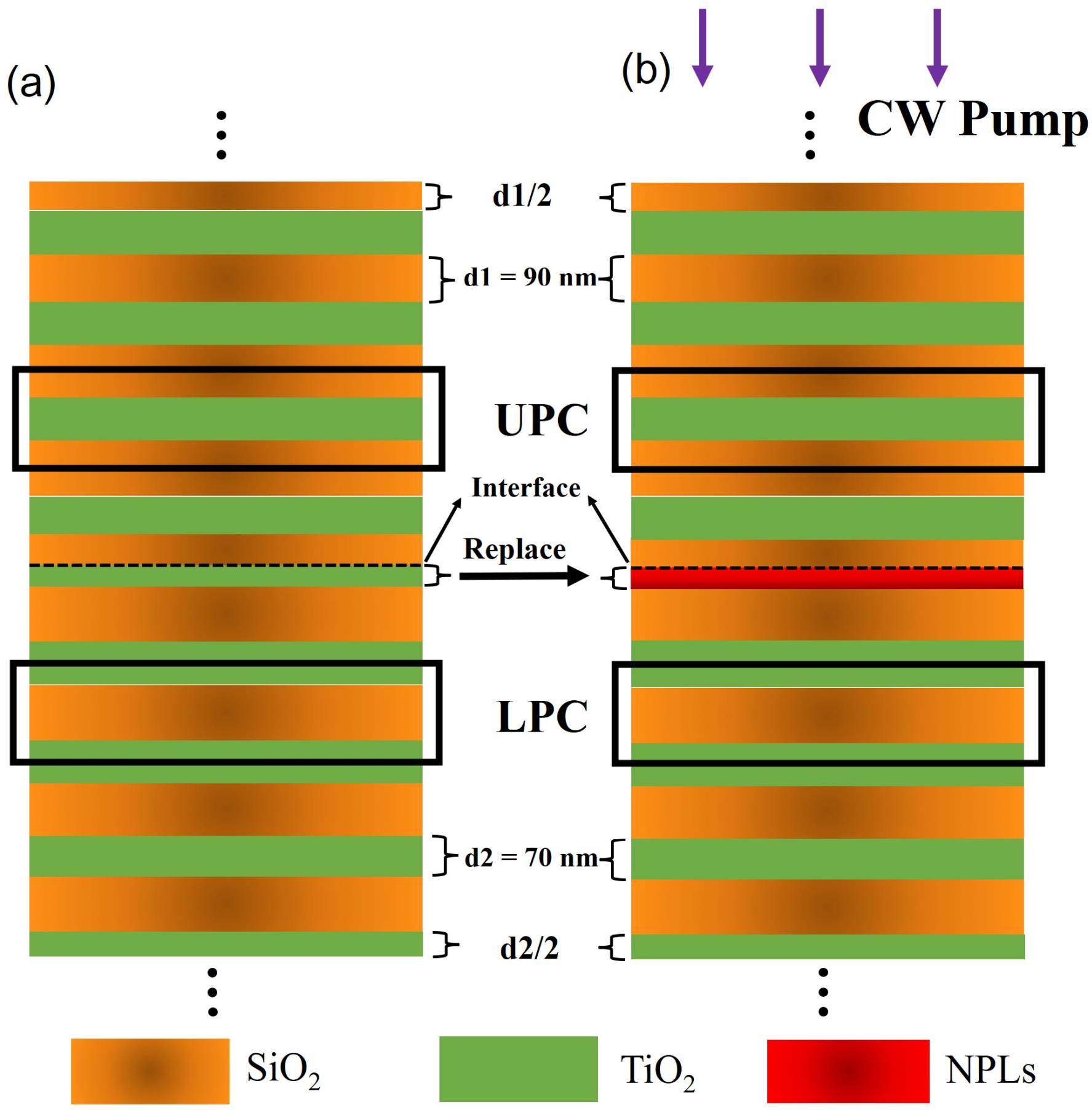

Fig. S23. Topological vertical-cavity design. (a) Schematic illustration of the topological vertical cavity composed of alternating SiO_2 (90 nm, orange) and TiO_2 (70 nm, green) layers. The unit cell of the upper photonic crystal (UPC) is SiO_2 (45 nm)–TiO_2 (70 nm)–SiO_2 (45 nm), whereas that of the lower photonic crystal (LPC) is TiO_2 (35 nm)–SiO_2 (90 nm)–TiO_2 (35 nm).(b) To form the topological laser cavity, the TiO_2 layer (35 nm) at the interface is replaced with CdSe CNPs. Owing to the topological robustness of the cavity, variations in the CNP thickness do not compromise the strong optical confinement, enabling tunable lasing wavelengths.

The topological vertical-cavity laser was fabricated using a solution-processed self-assembly method. A cleaned topological DBR mirror was first placed in a Teflon well, followed by rapid deposition of an alkane CNP solution onto the subphase surface. After solvent evaporation, the subphase was slowly drained through a needle to transfer a closely packed and uniformly oriented CNP thin film onto the DBR surface. This process could be repeated to obtain the desired CNP-layer thickness. Finally, a

complementary topological DBR mirror was bonded to the structure using optical glue applied along the periphery. The detailed fabrication procedure and topological mechanism follow previously published work *(6)*.

Table S3. Comparison of tunability among representative tunable laser systems.

Laser system	Gain / tuning mechanism	Spectral region	Tuning range
**This work: single CNP gain medium**	CdSe/CdSeS/CdZnS CNPs + external cavity	Visible	~50 nm
**This work: multiple CNP gain media**	Band-engineered CNPs + external cavity	Visible	~170 nm
**Visible ECDL**	Semiconductor gain chip + external cavity	Visible	Several–tens of nm
**Dye laser**	Dye solution + cavity tuning	UV–visible	Tens–hundreds of nm
**Ti:sapphire laser**	Ti:sapphire crystal	Near-IR, ~680–1080 nm	~ 400 nm
**OPO-based laser**	Nonlinear frequency conversion	Visible–IR	Hundreds of nm or broader

ECDL, external-cavity diode laser; UV, ultraviolet; IR, infrared; OPO, optical parametric oscillator

The tuning range of the present CNP liquid laser is defined as ~50 nm for a single CdSe/CdSeS/CdZnS core/crown/shell gain medium and ~170 nm across multiple band-engineered CNP gain media. Compared with visible ECDLs, the present platform offers a relatively large visible-band tuning range while retaining stable single-frequency operation and liquid-phase gain reconfigurability.

Table S4. Spectral-performance comparison of solution-processed coherent sources

Laser system	Gain medium	Spectral region / tunability	Linewidth	SMSR	long-term stability	Output intensity stability
**This work**	CdSe/CdSeS/ CdZnS CNPs	~50.3 nm for one medium; >170 nm across multiple media	0.051 nm	>31 dB	over 72 h (without circulation)	＜0.006
(21)	Type-(I+II) CQDs	575–634 nm	0.12 nm	—	5 h	—
(20)	ZnSe–ZnS CQDs	~417 nm – ~424 nm	<0.2 nm	—	6 h	—
(22)	CdSe/ZnSe/ ZnSeS/ZnS CQDs	Multicolor liquid lasing	~0.08 nm	—	50 h	—
(58)	$CsPbBr_3$ CQDs, $CsPbI_3$ CQDs	Green and deep-red liquid lasing	~0.1 nm	—	more than 10 days (with circulation)	—
(25)	CdZnSe/ZnSeS /ZnS CQDs	deep blue to red	~ 4.5 nm	—	50 h	—

REFERENCES

1. Y. Y. Jiang, A. D. Ludlow, N. D. Lemke, R. W. Fox, J. A. Sherman, L.-S. Ma, C. W. Oates, Making optical atomic clocks more stable with 10^{-16}-level laser stabilization. *Nat. Photonics* **5**, 158–161 (2011).

2. T. Kessler, C. Hagemann, C. Grebing, T. Legero, U. Sterr, F. Riehle, M. J. Martin, L. Chen, J. Ye, A sub-40-mHz-linewidth laser based on a silicon single-crystal optical cavity. *Nat. Photonics* **6**, 687–692 (2012).

3. M. Schioppo, J. Kronjäger, A. Silva, R. Ilieva, J. W. Paterson, C. F. A. Baynham, W. Bowden, I. R. Hill, R. Hobson, A. Vianello, M. Dovale-Álvarez, R. A. Williams, G. Marra, H. S. Margolis, A. Amy-Klein, O. Lopez, E. Cantin, H. Álvarez-Martínez, R. Le Targat, P. E. Pottie, N. Quintin, T. Legero, S. Häfner, U. Sterr, R. Schwarz, S. Dörscher, C. Lisdat, S. Koke, A. Kuhl, T. Waterholter, E. Benkler, G. Grosche, Comparing ultrastable lasers at 7×10^{-17} fractional frequency instability through a 2220 km optical fibre network. *Nat. Commun.* **13**, 212 (2022).

4. D. Psaltis, S. R. Quake, C. Yang, Developing optofluidic technology through the fusion of microfluidics and optics. *Nature* **442**, 381–386 (2006).

5. T. E. Drake, J. R. Stone, T. C. Briles, S. B. Papp, Thermal decoherence and laser cooling of Kerr microresonator solitons. *Nat. Photonics* **14**, 480–485 (2020).

6. R. Duan, Q. Zhang, Y. T. Thung, X. Zhou, T. Yin, Y. Ao, L. Xiao, Z. Zhang, C. X. X. Lee, T. Ren, H. V. Demir, W. S. Lew, B. Zhang, H. Sun, Continuous-wave pumped self-assembled colloidal topological lasers. *Adv. Mater.* **37**, 2416635 (2025).

7. R. Duan, Z. Zhang, L. Xiao, X. Zhao, Y. T. Thung, L. Ding, Z. Liu, J. Yang, V. D. Ta, H. Sun, Ultralow-threshold and high-quality whispering-gallery-mode lasing from colloidal core/hybrid-shell quantum wells. *Adv. Mater.* **34**, 2108884 (2022).

8. S. Ithurria, M. D. Tessier, B. Mahler, R. P. S. M. Lobo, B. Dubertret, A. L. Efros, Colloidal nanoplatelets with two-dimensional electronic structure. *Nat. Mater.* **10**, 936–941 (2011).

9. P. Geiregat, C. Rodá, I. Tanghe, S. Singh, A. Di Giacomo, D. Lebrun, G. Grimaldi, J. Maes, D. Van Thourhout, I. Moreels, A. J. Houtepen, Z. Hens, Localization-limited exciton oscillator strength in colloidal CdSe nanoplatelets revealed by the optically induced Stark effect. *Light Sci. Appl.* **10**, 112 (2021).

10. J. Yu, S. Hou, M. Sharma, L. Y. M. Tobing, Z. Song, S. Delikanli, C. Hettiarachchi, D. Zhang, W. Fan, M. D. Birowosuto, H. Wang, H. V. Demir, C. Dang, Strong plasmon-Wannier Mott exciton interaction with high aspect ratio colloidal quantum wells. *Matter* **2**, 1550–1563 (2020).

11. B. Guzelturk, M. Pelton, M. Olutas, H. V. Demir, Giant modal gain coefficients in colloidal II–VI nanoplatelets. *Nano Lett.* **19**, 277–282 (2019).

12. F. Isik, S. Delikanli, E. G. Durmusoglu, A. T. Isik, F. Shabani, H. Dehghanpour Baruj, H. V. Demir, "Giant" colloidal quantum well heterostructures of CdSe@CdS core@shell nanoplatelets from 9.5 to 17.5 monolayers in thickness enabling ultra-high gain lasing. *Small* **20**, 2309494 (2024).

13. C. E. Rowland, I. Fedin, H. Zhang, S. K. Gray, A. O. Govorov, D. V. Talapin, R. D. Schaller, Picosecond energy transfer and multiexciton transfer outpaces Auger recombination in binary CdSe nanoplatelet solids. *Nat. Mater.* **14**, 484–489 (2015).

14. J. Yu, C. Dang, Colloidal metal chalcogenide quantum wells for laser applications. *Cell Rep. Phys. Sci.* **2**, 100308 (2021).

15. R. Duan, Y. T. Thung, Y. He, G. Wei, Y. Wang, L. Xiao, Z. Zhang, T. Ren, L. Zhang, V. D. Ta, H. Sun, Multiwavelength, ultranarrow line width emission from fiber-capillary-integrated colloidal quantum well lasers. *ACS Nano* **19**, 26514–26524 (2025).

16. R. Duan, Y. T. Thung, Z. Zhang, E. G. Durmusoglu, Y. He, L. Xiao, C. X. X. Lee, W. S. Lew, L. Zhang, H. Li, J. Yang, H. V. Demir, H. Sun, Colloidal nanoplatelets-based soft matter technology for photonic interconnected networks: Low-threshold lasing and polygonal self-coupling microlasers. *Laser Photonics Rev.* **18**, 2300745 (2024).

17. F. Freire-Fernández, N. G. Sinai, M. J. H. Tan, S.-M. Park, E. Koessler, T. D. Krauss, P. Huo, T. W. Odom, Room-temperature polariton lasing from CdSe core-only nanoplatelets. *ACS Nano* **18**, 15177–15184 (2024).

18. T. T. H. Do, Z. Yuan, E. G. Durmusoglu, H. K. Shamkhi, V. Valuckas, C. Zhao, A. I. Kuznetsov, H. V. Demir, C. Dang, H. S. Nguyen, S. T. Ha, Room-temperature lasing at flatband bound states in the continuum. *ACS Nano* **19**, 19287–19296 (2025).

19. N. Vu Cam, M. A. Rahman, A. Syed, E. G. Durmusoglu, T. T. H. Do, P. L. Hernandez-Martinez, C. Dabard, D. Arora, S. Uddin, G. Zamiri, H. Wang, S. T. Ha, N. A. Mortensen, H. V. Demir, J. K. W. Yang, Tunable multiresonant microcavity exciton-polaritons in colloidal quantum wells. *Nano Lett.* **25**, 6109–6116 (2025).

20. X. Lin, Y. Yang, X. Li, Y. Lv, Z. Wang, J. Du, X. Luo, D. Zhou, C. Xiao, K. Wu, Blue lasers using low-toxicity colloidal quantum dots. *Nat. Nanotechnol.* **20**, 229–236 (2025).

21. D. Hahm, V. Pinchetti, C. Livache, N. Ahn, J. Noh, X. Li, J. Du, K. Wu, V. I. Klimov, Colloidal quantum dots enable tunable liquid-state lasers. *Nat. Mater.* **24**, 48–55 (2025).

22. X. Li, J. Du, Y. Jia, S. Hu, G. Li, B. Zhang, J. Zhu, J. Guo, K. Wu, Stable, efficient, and scalable multicolor lasing from colloidal quantum dots in liquids. *ACS Nano* **19**, 27488–27499 (2025).

23. Q. Zhang, R. Duan, Y. Ao, L. Wang, X. Zhou, X. Yang, Q. Zhang, X.-C. Yuan, B. Zhang, H. Sun, Nanoimprinted topological laser in the visible. *Sci. Bull.* **71**, 1941–1948 (2026).

24. R. Duan, B. Xu, Y. Wang, Y. He, H. Wang, G. Wei, Y. Wang, L. Wang, K. Lu, Y. Shi, X. Yang, J. Qi, H. Sun, Facile mode selection in colloidal quantum well lasers for ultralow-threshold reconfigurable single-/multi-mode operation. *ACS Photonics* **13**, 2721–2729 (2026).

25. Z. Huang, Y. Ren, Y. Wu, J. Qin, K. Jiao, H. Zhang, V. D. Ta, F. Gao, H. Shen, Y. Wang, Sustained continuous-wave lasing in quantum dot microfluids. *Adv. Mater.* **37**, 2509457 (2025).

26. H. Zhang, K. Jiao, Y. Hu, Z. Huang, Y. Wu, Q. Song, V. D. Ta, H. Sun, H. Shen, Y. Wang, A new generation of multimilliwatt-class colloidal quantum-dot lasers at full colors. *Adv. Mater.* **37**, 2413828 (2025).

27. Y.-S. Park, J. Roh, B. T. Diroll, R. D. Schaller, V. I. Klimov, Colloidal quantum dot lasers. *Nat. Rev. Mater.* **6**, 382–401 (2021).

28. N. Ahn, C. Livache, V. Pinchetti, V. I. Klimov, Colloidal semiconductor nanocrystal lasers and laser diodes. *Chem. Rev.* **123**, 8251–8296 (2023).

29. Y. Zeng, W. Yu, Y. Liu, W. Chen, Q. Wang, F. Cao, J. Wei, F. Liu, X. Yang, H. Li, Improved efficiency and stability in pure-red CdSe nanoplatelet LEDs enabled by gradient alloyed CdSeS/CdZnS crown/shell. *Adv. Mater.* **37**, 2415569 (2025).

30. T. Yin, X. Liang, Y. Huang, Y. T. Thung, Z. Ni, H. Sun, H. V. Demir, Engineering colloidal quasi-2D quantum wells for high-performance room-temperature single-photon sources. *J. Am. Chem. Soc.* **147**, 34540–34547 (2025).

31. V. I. Klimov, A. A. Mikhailovsky, S. Xu, A. Malko, J. A. Hollingsworth, C. A. Leatherdale, H.-J. Eisler, M. G. Bawendi, Optical gain and stimulated emission in nanocrystal quantum dots. *Science* **290**, 314–317 (2000).

32. R. R. Cooney, S. L. Sewall, D. M. Sagar, P. Kambhampati, Gain control in semiconductor quantum dots via state-resolved optical pumping. *Phys. Rev. Lett.* **102**, 127404 (2009).

33. P. Papagiorgis, A. Manoli, S. Michael, C. Bernasconi, M. I. Bodnarchuk, M. V. Kovalenko, A. Othonos, G. Itskos, Unraveling the radiative pathways of hot carriers upon intense photoexcitation of lead halide perovskite nanocrystals. *ACS Nano* **13**, 5799–5809 (2019).

34. J. Chen, K. Žídek, P. Chábera, D. Liu, P. Cheng, L. Nuuttila, M. J. Al-Marri, H. Lehtivuori, M. E. Messing, K. Han, K. Zheng, T. Pullerits, Size- and wavelength-dependent two-photon absorption cross-section of $CsPbBr_3$ perovskite quantum dots. *J. Phys. Chem. Lett.* **8**, 2316–2321 (2017).

35. V. I. Klimov, A. A. Mikhailovsky, D. W. McBranch, C. A. Leatherdale, M. G. Bawendi, Quantization of multiparticle Auger rates in semiconductor quantum dots. *Science* **287**, 1011–1013 (2000).

36. K. Wu, Y.-S. Park, J. Lim, V. I. Klimov, Towards zero-threshold optical gain using charged semiconductor quantum dots. *Nat. Nanotechnol.* **12**, 1140–1147 (2017).

37. B. R. C. Vale, E. Socie, A. Burgos-Caminal, J. Bettini, M. A. Schiavon, J.-E. Moser, Exciton, biexciton, and hot exciton dynamics in $CsPbBr_3$ colloidal nanoplatelets. *J. Phys. Chem. Lett.* **11**, 387–394 (2020).

38. J. J. Geuchies, R. Dijkhuizen, M. Koel, G. Grimaldi, I. du Fossé, W. H. Evers, Z. Hens, A. J. Houtepen, Zero-threshold optical gain in electrochemically doped nanoplatelets and the physics behind it. *ACS Nano* **16**, 18777–18788 (2022).

39. Q. Li, Z. Xu, J. R. McBride, T. Lian, Low-threshold multiexciton optical gain in colloidal CdSe/CdTe core/crown type-II nanoplatelet heterostructures. *ACS Nano* **11**, 2545–2553 (2017).

40. Q. Li, T. Lian, A model for optical gain in colloidal nanoplatelets. *Chem. Sci.* **9**, 728–734 (2018).

41. A. V. Malko, A. A. Mikhailovsky, M. A. Petruska, J. A. Hollingsworth, V. I. Klimov, Interplay between optical gain and photoinduced absorption in CdSe nanocrystals. *J. Phys. Chem. B* **108**, 5250–5255 (2004).

42. Y. Wu, Z. Huang, Q. Sun, V. D. Ta, S. Wang, Y. Wang, A new generation of liquid lasers from engineered semiconductor nanocrystals with giant optical gain. *Laser Photonics Rev.* **17**, 2200703 (2023).

43. Y. Wu, W. Cai, Z. Huang, Y. Ren, Y. Wu, Y. Wang, An all-colloidal and eco-friendly quantum-dot laser. *Laser Photonics Rev.* **18**, 2301167 (2024).

44. F. Fan, O. Voznyy, R. P. Sabatini, K. T. Bicanic, M. M. Adachi, J. R. McBride, K. R. Reid, Y.-S. Park, X. Li, A. Jain, R. Quintero-Bermudez, M. Saravanapavanantham, M. Liu, M.

Korkusinski, P. Hawrylak, V. I. Klimov, S. J. Rosenthal, S. Hoogland, E. H. Sargent, Continuous-wave lasing in colloidal quantum dot solids enabled by facet-selective epitaxy. *Nature* **544**, 75–79 (2017).

45. H. Shi, X. Zhang, X. Sun, R. Chen, X. Zhang, Direct and indirect recombination and thermal kinetics of excitons in colloidal all-inorganic lead halide perovskite nanocrystals. *J. Phys. Chem. C* **123**, 19844–19850 (2019).

46. M. Pelton, Y. Wang, I. Fedin, D. V. Talapin, S. K. O'Leary, Hot-carrier relaxation in CdSe/CdS core/shell nanoplatelets. *J. Phys. Chem. C* **124**, 1020–1026 (2020).

47. Y. Min, M. Akbulut, K. Kristiansen, Y. Golan, J. Israelachvili, The role of interparticle and external forces in nanoparticle assembly. *Nat. Mater.* **7**, 527–538 (2008).

48. K. J. M. Bishop, C. E. Wilmer, S. Soh, B. A. Grzybowski, Nanoscale forces and their uses in self-assembly. *Small* **5**, 1600–1630 (2009).

49. M. Grzelczak, J. Vermant, E. M. Furst, L. M. Liz-Marzán, Directed self-assembly of nanoparticles. *ACS Nano* **4**, 3591–3605 (2010).

50. Y. Sahoo, M. Cheon, S. Wang, H. Luo, E. P. Furlani, P. N. Prasad, Field-directed self-assembly of magnetic nanoparticles. *J. Phys. Chem. B* **108**, 3380–3383 (2004).

51. A. Ashkin, J. M. Dziedzic, J. E. Bjorkholm, S. Chu, Observation of a single-beam gradient force optical trap for dielectric particles. *Opt. Lett.* **11**, 288–290 (1986).

52. A. Ashkin, Optical trapping and manipulation of neutral particles using lasers. *Proc. Natl. Acad. Sci. U.S.A.* **94**, 4853–4860 (1997).

53. K. C. Neuman, S. M. Block, Optical trapping. *Rev. Sci. Instrum.* **75**, 2787–2809 (2004).

54. M. G. Donato, O. Brzobohatý, S. H. Simpson, A. Irrera, A. A. Leonardi, M. J. Lo Faro, V. Svak, O. M. Maragò, P. Zemánek, Optical trapping, optical binding, and rotational dynamics of silicon nanowires in counter-propagating beams. *Nano Lett.* **19**, 342–352 (2019).

55. B. Shi, S. Xing, Y. Zhang, Y. Gao, W. Gao, X. Li, H. Li, Q. Jia, R. Feng, D. Tang, T. Zhu, F. Sun, Y. Cao, W. Ding, Topological slow light enabled huge optical force enhancement. *ACS Photonics* **11**, 4414–4421 (2024).

56. E. S. Ferre-Pikal, J. R. Vig, J. C. Camparo, L. S. Cutler, L. Maleki, W. J. Riley, S. R. Stein, C. Thomas, F. L. Walls, J. D. White, Draft revision of IEEE Std 1139–1988 standard definitions of physical quantities for fundamental frequency and time metrology—random instabilities, in *Proceedings of the 1997 IEEE International Frequency Control Symposium* (IEEE, 1997), pp. 338–357.5656

57. E. Loth, Drag of non-spherical solid particles of regular and irregular shape. *Powder Technol.* **182**, 342–353 (2008).

58. X. Li, Y. Jia, M. Liu, S. He, J. Guo, K. Wu, Ultrastable lasing from perovskite colloidal nanocrystals. *Sci. Adv.* **11**, eadq9002 (2025).
